# Complications of XEN gel stent implantation for the treatment of glaucoma: a systematic review

**DOI:** 10.3389/fmed.2024.1360051

**Published:** 2024-05-06

**Authors:** Lu Gan, Lixiang Wang, Jun Chen, Li Tang

**Affiliations:** ^1^Department of Ophthalmology, West China Hospital, Sichuan University, Chengdu, China; ^2^Department of Ophthalmology, West China School of Public Health and West China Fourth Hospital, Sichuan University, Chengdu, China

**Keywords:** XEN gel stent, glaucoma, complications, surgery, XEN 45 gel stent, review

## Abstract

**Aim:**

This study was aimed to summarize the complications and their management associated with XEN gel stent implantation.

**Methods:**

A systematic review of literature was conducted using Medline (via PubMed), EMBASE, the Cochrane Library databases, and China National Knowledge Infrastructure, from their inception to February 1, 2024.

**Results:**

A total of 48 studies published between 2017 and 2024 were identified and included in the systematic review, including 16 original studies (retrospective or prospective clinical studies), 28 case reports, and 4 case series, which followed patients for up to 5 years. Early postoperative complications of XEN gel stent implantation include hypotony maculopathy (1.9–4.6%), occlusion (3.9–8.8%), suprachoroidal hemorrhage (SCH), choroidal detachment (0–15%), conjunctival erosion, and exposure of the XEN gel stent (1.1–2.3%), wound and bleb leaks (2.1%) and malignant glaucoma (MG) (2.2%). Mid-postoperative complications of XEN gel stent implantation included migration of XEN (1.5%), ptosis (1.2%), endophthalmitis (0.4–3%), macular edema (1.5–4.3%), hypertrophic bleb (8.8%) and subconjunctival XEN gel stent fragmentation (reported in 2 cases). Late postoperative complications reported in cases included spontaneous dislocation and intraocular degradation.

**Conclusion:**

XEN gel stent implantation is a minimally invasive glaucoma surgery (MIGS) procedure for glaucoma, known for its potential to minimize tissue damage and reduce surgical duration. However, it is crucial to note that despite these advantages, there remains a risk of severe complications, including endophthalmitis, SCH, and MG. Therefore, postoperative follow-up and early recognition of severe complications are essential for surgical management.

## Introduction

Glaucoma is the leading cause of irreversible blindness, and an estimated 111.8 million people aged 40–80 years old will be affected by glaucoma globally in 2040 (1, 2). The only confirmed modifiable glaucoma risk factor is elevated intraocular pressure (IOP). Multiple methods to reduce IOP have been explored and verified, including medications, laser, and surgery, with new therapies continuously revolutionizing glaucoma treatment (3). Trabeculectomy is a classical surgical method for glaucoma and has been the standard procedure in many medical centers for decades, with sound evidence supporting its long-term efficacy and safety (4, 5). However, severe complications, including malignant glaucoma (MG), bleb-related infection, and expulsive choroidal hemorrhage (6), can occur.

The recent development of new devices that are significantly less invasive, collectively termed minimally invasive glaucoma surgery (MIGS), offers a new perspective on reducing IOP with lower risk, shorter operating times, and faster recovery (7). It is performed using a less invasive “*ab interno*” approach, which reduces damage to surrounding tissues and preserves the conjunctiva (8, 9). Currently, the XEN gel stent (Allergan PLC, Irvine, CA, United States) has been the only MIGS device that allows subconjunctival filtration and has been used to treat open-angle glaucoma (10). However, it has also been reported in some case series to be effective in treating angle closure glaucoma (11), uveitic glaucoma (12), neovascular glaucoma (13), iridocorneal endothelial (ICE) syndrome (14), and steroid-induced glaucoma (15). The XEN gel stent comes in three different diameters (45, 63, and 140 μm) to provide varying levels of IOP control.

**Figure 1:**
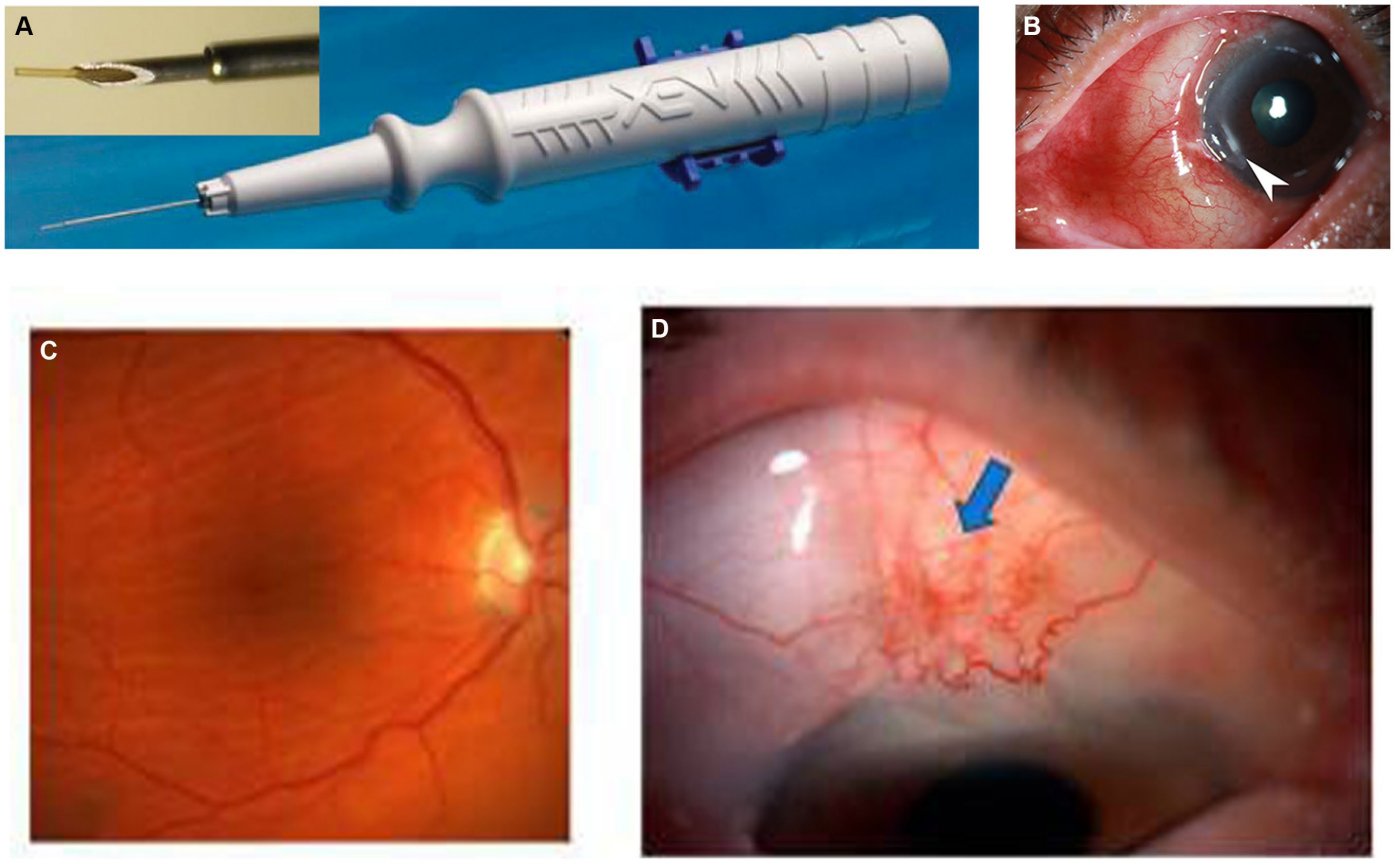
**(A)** Grover et al. (17). **(B)** Hypertrophic bleb. **(C,D)** Kosior-Jarecka et al. (18).

The XEN45 is a tubular implant with a total length of 6 mm and an inner diameter of 45 μm, made of cross-linked porcine gelatin, a type of hydrophilic collagen. The implant is rigid when dry and becomes soft within 1–2 min when hydrated, adapting to the tissue shape, thus preventing migration and potential erosion. Studies have shown that the gel stent is approximately 100 times more flexible than the silicone tubing used in traditional tube–shunt surgery (16). The implant is housed in a disposable preloaded handheld inserter designed specifically for *ab interno* surgical implantation (7) (Figure 1A). It was designed based on principles of laminar fluid dynamics (Hagen–Poiseuille equation) to prevent early postoperative hypotony, as demonstrated by recent experimental studies (19). The rate of aqueous humor turnover is estimated to be 1.0–1.5% of the anterior chamber (AC) volume per minute, which is 2.4 ± 0.6 μL/min (mean ± standard deviation [SD], daytime measurements in adults aged 20–83 years), and the XEN45 provides a flow of 1.2 μL/min (at a 5 mmHg pressure gradient), offering approximately 6–8 mmHg flow resistance, which reduces the risk of hypotony (16).

The *ab interno* procedure involves inserting the Xen Gel Stent through a clear corneal incision, positioning it within the trabecular meshwork and extending it into the subconjunctival space. By creating a new drainage pathway that bypasses the trabecular meshwork, the Xen implant facilitates aqueous humor outflow from the anterior chamber to the subconjunctival space, where it is absorbed by the surrounding tissue (20). XEN gel stent implantation directly drains aqueous humor from the AC to the subconjunctival space, bypassing the resistance of the dysfunctional trabecular meshwork (16). XEN gel stent implantation has been reported to provide up to 56% reduction in IOP and a decrease in the average number of medications used by 2.7 at 12 months (21). Furthermore, it has a lower complication rate compared to conventional trabeculectomy (22). The advantages of XEN gel stent implantation include minimally invasive access through *ab interno*/*ab externo* approaches, preservation of the sclera and conjunctiva, better preservation of corneal endothelium, elimination of the need for iridectomy and sutures, and shorter surgery time (23). Additionally, XEN gel stent implantation can be performed alone or concurrently with cataract surgery (24). General recommendations for preoperative assessment, surgical technique, and postoperative follow-up of XEN gel stent implantation have been published (25). However, despite numerous clinical studies and case reports providing relevant information, a comprehensive summary of complications associated with this surgical method, especially rare ones, has not been compiled. In this systematic review, we aim to comprehensively summarize all complications of XEN 45 gel stent implantation, including their incidence, risk factors, available treatments, and preventive measures.

## Methods

**Figure 2:**
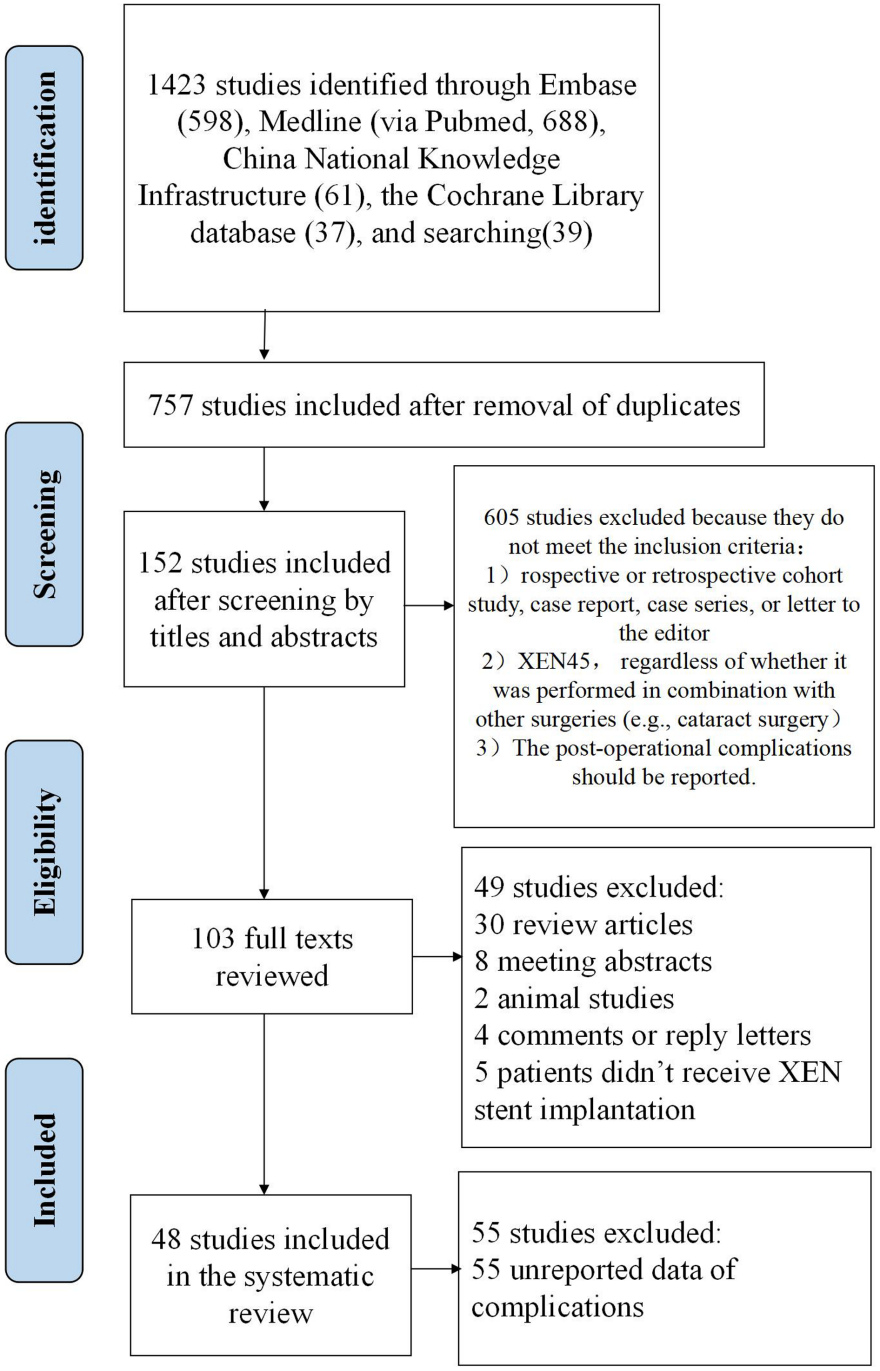
The flow diaphragm of study selection for the systematic review.

Following the guidelines of the Preferred Reporting Items for Systematic Reviews and Meta-analyses (PRISMA) and the Meta-analysis of Observational Studies in Epidemiology (MOOSE) (26), a comprehensive search of Medline (via Pubmed), EMBASE, the Cochrane Library databases, and China National Knowledge Infrastructure were performed from their conception to February 1st, 2024. The search was independently performed by two investigators, Lu Gan and Lixiang Wang, using a combination of keywords in English or their corresponding Chinese terms, including “XEN,” “glaucoma,” “micro-stent,” “gel implant,” and “MIGS.” In addition to the computer-assisted search, the references in the articles retrieved from this bibliographic search were also manually searched and studied. The detailed steps and results of the search strategy are presented in Figure 2.

Studies published in English or Chinese were included in the review. The following criteria were used to select studies for the systematic review: (1) The study should be a prospective or retrospective cohort study, case report, case series, or letter to the editor. Reviews, editorial materials, and meeting abstracts were not included. (2) The surgery of interest should be XEN45 gel stent implantation, regardless of whether it was performed in combination with other surgeries (e.g., cataract surgery). (3) The post-operational complications should be reported. Exclusion criteria were as follows: (1) Animal studies. (2) Incomplete report of surgical complications or lack of essential data for analysis. (3) XEN63. (4) XEN140.

The data extracted from the included studies included author names, publication year, publication types, number of patients, age, sex, surgical method, surgical complications, and follow-up results. The data extraction process was independently conducted by two reviewers, Lu Gan and Lixiang Wang.

## Systematic review

A total of 48 studies published between 2017 and 2024 were identified and included in the systematic review, including 16 original studies (retrospective or prospective clinical studies), 28 case reports, and 4 case series. Patients were followed for up to 5 years after XEN gel stent implantation. Below, we provided a summary of perioperative complications, their mechanisms, and management. A brief summary of surgical complications is listed in Table 1.

**Table 1:** Summary of surgical complications of XEN gel stent implantation.

Complications	Incidence	Onset time	Management	References
Early postoperative period (0–30 days)				
Occlusion	3.9–8.8%	Different times after surgery	Nd: YAG laser ablation, intracameral plasminogen activator, surgical removal	(27–34)
Hypotony maculopathy	1.9–4.6%	<1 week or > 1 month	Conservative management (transconjunctival compressive sutures, bleb injection of autologous blood or viscoelastic material, and intracameral injection of viscoelastic material or gas) Surgical management (transconjunctival flap suturing, excision of thin blebs and conjunctival advancement, patch grafting)	(18, 35–47)
Suprachoroidal hemorrhage	N.A.	Both intraoperatively and postoperatively	surgical intervention (either by choroidal tap or par plana vitrectomy)	(48–52)
Choroidal detachment	0–15%	1 week	Spontaneously resolve or use topical or oral steroids and cycloplegics.	(17, 27, 53–56)
Conjunctival erosion and exposure of XEN gel stent	1.1–2.3%	Early after the implantation surgery or months later	Conservative methods (pressure patching, wearing contact lenses, crosslinking, and application of vitamin A ointments) Surgical management conjunctival grafting, *ab interno* repositioning of XEN gel stent through the anterior chamber, and direct suturing of the conjunctival defect	(57–61)
Wound and bleb leaks	2.1%	2 weeks or more than 3 months	Conservative treatment (therapeutic contact lens, autologous blood injection, injection of fibrin glue, application of cryotherapy, and laser treatment) Surgical procedures (amniotic membrane transplantation, conjunctival autograft, and conjunctival sliding)	(61–65)
Malignant glaucoma	2.2%	4 days	Laser capsulotomy, hyaloidotomy, or iridotomy, or by surgical methods such as vitrectomy or posterior capsulotomy	(66–69)
Ptosis	1.2%	Early after surgery	Spontaneously resolve, but surgical management is needed for persistent ptosis	(62)
Mid-postoperative period (1–6 months)				
XEN gel stent migration	1.5%	4 months	Replace with a new XEN stent	(17, 70)
Endophthalmitis	0.4–3%	More than 3 months	Removal of exposed stent, topical, systemic, and bleb revision	(12, 27, 55, 71–73)
Macular edema	1.5–4.3%	≥1 month	Spontaneously resolve	(62, 74, 75)
Hypertrophic bleb	8.8%	Months to years after surgery	Conservative therapies (injection of autologous platelet concentrates, blockage with viscoelastic of the *ab-interno* stent, and sealing with a tissue adhesive) Surgical management (scleral fixation sutures, bleb revision surgery).	(76–80)
Subconjunctival XEN gel stent fragmentation	2 cases	2–3 months	Observation or replace with a new XEN stent	(81, 82)
Late postoperative period (from 6 months)				
Late spontaneous dislocation	One case	6 months	Replace with a new XEN stent	(83)
Intraocular degradation	One case	3 years	Replace with a new XEN stent	(84)

### Early postoperative period (0–30 days)

#### Occlusion of XEN

Occlusion of the XEN stent lumen is a relatively common complication that can occur at various times after surgery, with a reported incidence between 3.9 and 8.8% (27, 28). The occluding objects can be blood clots, fibrous scar tissue, or the iris (29, 85) due to the stent’s small internal lumen (29, 30, 86, 87). In Asian eyes with a relatively shallow AC and crowded anterior segment, there is an increased likelihood of anatomical obstruction of the XEN implant by the iris. Specialists have proposed several potential causes for this occlusion event, including the posterior positioning of the XEN gel stent opening, which results in a higher risk of iris occlusion; the short length of insertion into the AC; overactive filtration leading to local turbulence in the early postoperative period; and the patient habit of rubbing the surgical eye, resulting in shifting of the floppy iris. The approach to relieving occlusion depends on the etiology and nature of the occluding objects. For iris-related occlusion, argon laser peripheral iridoplasty or low energy neodymium-doped yttrium aluminum garnet (Nd:YAG) laser lysis of the blocking iris is commonly used to alleviate local occlusion (30). When the XEN gel stent implant is blocked with fibrin or blood clots, the occluding objects can be removed through intracameral injection of tissue plasminogen activator, ablation with Nd:YAG laser, or surgical clamping using internal limiting membrane forceps or vitreous scissors. Alternatively, surgery with a 10–0 nylon suture may be performed to recanalize the XEN gel stent (29, 31–34).

#### Hypotony maculopathy

HM is a relatively rare complication associated with XEN gel stent implantation that can occur within less than 1 week or more than 1 month, with a reported incidence of 1.9–4.6% (27, 62) (Figures 1C,D). Macular hypotony is characterized by a decrease in visual acuity caused by macular folds, retinal edema, papilledema, and vascular tortuosity. It is believed that low IOP levels cause thickening of the perifoveal choroid and sclera, resulting in their central displacement, which is visible as macular folds. Over time, these changes lead to photoreceptor damage and become irreversible, which may limit the recovery of visual function even after restoring normal IOP (35, 88). Several risk factors for hypotony after glaucoma surgery have been identified, including myopia, young age, the use of antimetabolites, pre-existing inflammation, aphakia, and old age accompanied by a thin conjunctiva and thinner central corneal thickness (36–38). Patients with high myopia are at a higher risk of developing ocular hypotony due to their thin scleral wall, which may result in potential leakage of aqueous humor from the scleral incision adjacent to the site of XEN gel stent insertion (39). Treatment options for hypotony after glaucoma surgery, mainly caused by over-filtrating blebs, include conservative management with topical autologous serum (40), transconjunctival compressive sutures (18), bleb injection of autologous blood (41) or viscoelastic material (42), as well as AC injection of viscoelastic material (43) or gas (44). Conservative management usually has minor and short-lasting effects. Surgical management involves transconjunctival flap suturing (45, 46), excision of thin blebs and conjunctival advancement (35), patch grafting using donor sclera (47, 89), donor cornea (90), as well as autologous conjunctiva (91, 92).

#### Suprachoroidal hemorrhage

SCH is characterized by the accumulation of blood in the potential space between the choroid and the sclera, originating from the long/short posterior ciliary artery. It is a potentially sight-threatening but rare complication of XEN gel stent implantation, which can occur both intraoperatively or postoperatively. A number of potential risk factors have been linked to the development of intra-/post-operative SCH, including high preoperative IOP, severe postoperative hypotony, aphakia, pseudophakia, anticoagulation, white race, prior intraocular surgery, low postoperative IOP, systemic hypertension, ischemic heart disease, as well as pulmonary disease (93, 94). The exact pathophysiology of SCH is not fully understood, but the increased preoperative IOP, sudden decreased IOP, and postoperative hypotony are accepted risk factors associated with this complication in XEN implantation (48). Several SCH patients have been reported to be related with XEN gel stent implantation, and some cases were complicated with retinal detachment requiring surgical interventions (49–52). Wang et al. reported an 86-year-old patient on rivaroxaban due to atrial fibrillation who developed “kissing” SCH 3 days after the surgery resulting from ocular hypotony, which resolved with prompt surgical drainage (49). A sudden decease in IOP within or post-surgery is identified as the most significant risk factor. Once SCH occurs, various methods should be used to control the bleeding and preserve eyesight alongside the eyeball. Greater emphasis should be placed on preventive measures, and prompt action should be taken to close the wound. If the bleeding cannot be controlled, the sclera can be incised at a distance of 8–10 mm behind the limbus, which may allow for salvage of the SCH (95, 96). However, whether visual outcomes of early surgical intervention (via choroidal tap/par plana vitrectomy) turn out better in contrast to those of conservative management is still under debate. Particularly for cases with limited SCH, a wait-and-see strategy for the spontaneous absorption of bleeding is a reasonable choice, but close follow-up is essential (51). For patients with known risk factors, including old age, anticoagulant use, and high IOP before surgery, SCH should be anticipated and promptly managed.

#### Choroidal detachment

CD is characterized by an abnormal accumulation of either blood or serum in the suprachoroidal space, which is located between the choroid and the sclera. Under physiological conditions, this space represents a potential volume owing to the close adjacency of the choroid and sclera. Under pathological conditions, fluid accumulation in this space may occur based on the changes in ocular fluid dynamics, specifically the equilibrium between hydrostatic and oncotic pressure. The incidence of CD following XEN gel stent implantation surgery varies from 0 to 15% in different studies and is considered a relatively common complication in some medical centers (17, 27, 53–56). The average time between XEN implantation and the onset of CD is approximately 1 week (56). Low post-operative IOP is a well-established risk factor for CD after glaucoma filtering surgeries. Patients who are taking multiple IOP-lowering medications before surgery are at an increased risk of developing CD after XEN gel stent implantation (97–99). The use of mitomycin C (MMC) may directly harm the ciliary epithelium, leading to decreased secretion. Additionally, the incidence of CD increases with age and is more frequent in the elderly, since their thin scleral wall makes the vortex veins susceptible to compression and leakage under increased venous pressure (56, 97, 99). The prognosis for CD is generally favorable, with most cases experiencing complete resolution within 5–30 days after onset. Patients may experience spontaneous resolution or use topical or oral steroids and cycloplegics as treatment options.

#### Conjunctival erosion and exposure of XEN gel stent

The erosion of the conjunctiva and extrusion of the XEN gel stent may occur shortly after the implantation surgery or months later. This mechanical complication has been reported in several studies (57, 100–102). Potential mechanisms for the initial conjunctival erosions include: (1) the administration of the anti-metabolite MMC; (2) the *ab interno* approach; (3) the subconjunctival position; (4) prolonged use of topical anti-glaucoma medications; as well as (5) mechanical stress, for instance, from elderly patients rubbing their eyes with their hands. The use of anti-metabolites can enhance the success rate of filtering surgery by mitigating the wound-healing process, yet it may increase the risk of bleb-related complications, like a thin-walled cystic bleb or surgically induced necrotizing scleritis (57, 58, 103). Since the XEN gel stent is implanted using the *ab interno* approach without the need for conjunctival dissection or sutures, there is a risk of malposition of the distal end of the implant. Continuous friction may then lead to conjunctival erosion and exposure of the stent (59). On the other hand, the application of topical anti-proliferative agents like MMC, to prevent conjunctival scarring and improve the success rate of glaucoma filtering surgery, is associated with the formation of thin-walled, cystic filtration blebs and may increase the risk of XEN gel exposure (104). Conservative methods, including pressure patching, wearing contact lenses, crosslinking, and application of vitamin A ointments remain the feasible way to manage bleb leakage (60, 61). When conservative methods fail, surgical management techniques such as the use of free conjunctival autograft, amniotic membrane graft, *ab interno* repositioning of the XEN gel stent through the AC, along with the direct suturing of the conjunctival defect have been shown to effectively repair the leaking conjunctiva and restore functional bleb (57–59). However, recurrent bleb leakage and exposure of the implant can still occur, making management challenging. Olate-Pérez et al. reported a case of managing a patient with conjunctival perforation that occurred 18 months after XEN gel stent implantation. The stent broke as the surgeon attempted to track the short distal end of the stent from the conjunctival side and was unable to be removed (57, 105). Therefore, caution should be taken during the implantation procedure, and the use of a fixation suture may be helpful, although further study is needed.

#### Bleb leakage

Bleb leakage is an uncommon but potentially sight-threatening complication of XEN gel stent implantation, with a reported incidence of 2.1% (62). Persistent conjunctival bleb leakage may cause over-infiltration and ocular hypotony, increasing the risk of infection, stent displacement, and endophthalmitis. During the surgery, the application of adjunctive antifibrotic agents such as mitomycin-C is responsible for creating a thin-walled perilimbal bleb that is prone to erosion and leakage. Additional risk factors consist of bleb manipulation, laser suturolysis, needling, or injection of autologous serum (63). In addition, some surgeons have found that dislocation and inappropriate positioning of the external part of the XEN gel stent, which directly rubs against the overlying conjunctiva, causes conjunctival erosion and subsequent leakage (106). The management of a leaking bleb includes conservative measures such as therapeutic contact lens as well as non-surgical treatments (autologous blood injection, fibrin glue injection, cryotherapy application, or laser treatment of the leaking bleb). Surgical procedures, including amniotic membrane transplantation, conjunctival autograft, as well as conjunctival sliding, can also be adopted (64). Conjunctival autograft can repair the leaking bleb, but sometimes replacement of the dislocated stent is required. In a case reported by Salinas et al., a 72-year-old female patient with bilateral pseudoexfoliation glaucoma and cataract received XEN gel stent implantation and phacoemulsification for both eyes at a one-week interval. Bleb leakage and exposure of the XEN gel stent occurred early after surgery at 2 weeks, which was managed by implantation of a new XEN gel stent and ab-externo bleb revision with removal of the old XEN gel stent (107). Surgeons need to carefully check the correct position of the stent during surgery to reduce the risk of direct conjunctival erosion. Some research suggests that a single session of crosslinking for a thin-filtered bleb with leakage following an episode of blebitis has demonstrated efficacy in resolving the bleb leakage (61, 63–65). The objective of employing crosslinking in a thin-walled leaking bleb is to promote the formation of covalent bonds in the collagen fibers of the conjunctival wall of the bleb, thereby enhancing its rigidity and resistance to rupture, reducing permeability, and thus preventing leakage (64). However, in instances where large holes are unlikely to seal, surgical management must be considered the preferred treatment option.

#### MG

MG is an uncommon but severe complication associated with all glaucoma surgeries. It presents with flattening of the central and peripheral AC and increased IOP with secondary angle closure. MG can develop early after surgery or years later and can occur in phakic, aphakic, or pseudophakic eyes (108). Schlenker et al. reported the incidence of MG to be 2.2% in 187 patients undergoing XEN gel stent implantation with the application of MMC, similar to other types of glaucoma surgeries for angle closure glaucoma (66, 67). It can be difficult to identify MG early in its course before an increase in IOP develops. Ultrasound biomicroscopy of the eyes during a MG episode reveals anterior rotation of the ciliary processes that press against the lens equator and limit the normal flow through the AC (109). The mechanisms associated with MG are not fully understood. The misdirection of the aqueous humor backward into the vitreous cavity and the forward displacement of the lens-iris diaphragm are recognized etiologies for the development of MG (69). Managing MG is challenging. The goal of medical treatment is to decrease aqueous humor production and vitreous shrinkage while concurrently reducing resistance in the channel of aqueous humor flow into the AC. The current acceptable conservative treatment regimen includes applying atropine, phenylephrine, blockers, and acetazolamide locally, as well as administering a 50% glycerol solution orally and mannitol intravenously. Local corticosteroids help reduce the associated inflammatory process. If improvement is achieved, the dosage of hyperosmotic agents can be decreased, followed by carbonic anhydrase inhibitors. However, mydriatic cycloplegic medications should be continued (109). Nevertheless, it has been reported that symptoms of MG tend to reappear when drugs are discontinued or modified. Therefore, medical treatment is considered temporary and is used in conjunction with laser iridotomy, posterior capsulotomy, and hyaloidotomy. However, currently, only one case report has discussed the management of MG associated with XEN gel stent implantation, which is similar to other surgeries (68). The key aim is to disrupt the anterior displacement of the iris-lens diaphragm, either by laser capsulotomy, hyaloidotomy, or iridotomy, or by surgical methods such as vitrectomy or posterior capsulotomy (69). However, the prognosis of MG and its risk factors associated with XEN gel stent implantation are largely unknown.

#### Ptosis

Ptosis is a relatively rare complication associated with XEN gel stent implantation, with a reported incidence of 1.2% (62). Some proposed mechanisms for the cause of ptosis after surgery include lid edema from locally administered anesthetic, initial myotoxic effects, and compression of the upper eyelid against the orbital bones from the eyelid speculum, which reduces blood flow to the levator muscle and contributes to the edema (110, 111). Causes of temporary ptosis are believed to include eyelid edema, indirect infiltration of the LPS by retrobulbar or peribulbar anesthesia, and ocular surface disturbance (112). Permanent postoperative ptosis is widely thought to be due to dehiscence of the LPS aponeurosis. The majority of ptosis cases develop early after surgery and may spontaneously resolve, but surgical management is necessary for persistent ptosis. Possible reasons for the higher incidence of ptosis associated with XEN gel stent implantation include levator aponeurosis injury with speculum use for wide opening of the palpebral fissure and the difficulty in washing out the toxicity of MMC and xylocaine compared to trabeculectomy. A “wait-and-see” strategy for transient ptosis resolution is reasonable, but further research is needed to explore possible ways to reduce the risk of ptosis.

### Mid-postoperative period (1–6 months)

#### Migration of XEN implant

The XEN gel stent implant is a highly flexible tube that easily conforms to the tissue shape and adopts an “S shape” when inserted into the AC through the scleral canal (16). However, if the XEN implant is not placed correctly, it can be affected by external forces such as blinking forces from the orbicularis muscle, friction, and micro-trauma, which may cause migration. Grover et al. reported an incidence of MG of 1.5% in 74 patients (17). As reported by Ali et al., when the XEN stent implant is placed deeper into the AC and the remaining length of the tube implanted under the subconjunctival space is less than 2 mm, the XEN implant becomes less flexible and its distal tips are angled obliquely, making it prone to dislocation under external forces (113). Therefore, it is recommended to carefully place the XEN implant with approximately 1 mm of visible insertion into the AC, approximately 3 mm of the exiting part out of the sclera, and 2 mm of the tube situated within the subconjunctival space (25). Prior to surgery, ensure appropriate treatment for patients with allergic eye disease to minimize eye rubbing and prevent stent migration as well as subsequent complications. While we cannot conclusively confirm eye rubbing as the primary trigger of this complication, it is probable that it played a role in the stent migration. Dervenis et al. presented a comparable case and proposed a modification in XEN stent design to prevent dislocation, for instance, a gradual increment in the lumen width (70).

#### Endophthalmitis

Endophthalmitis is a rare but potentially sight-threatening complication. Currently, only four studies have reported cases of endophthalmitis associated with XEN gel stent implantation. These cases occurred more than 3 months after surgery and were related to bleb complications (103, 114–116). The incidence of this condition is reported to be between 0.4 and 3% (27, 55, 71–73). Risk factors for bleb-related endophthalmitis include the use of anti-metabolites, a thin avascular bleb, bleb leakage, stent exposure, use of topical steroids, as well as young patient age (114). The common causative pathogens of bleb-related endophthalmitis are Streptococcus species, Moraxella, coagulase-negative Staphylococcus, and *Propionibacterium acnes* (117). Lim hypothesized that coinciding gastrointestinal infection and poor handwashing with stent exposure may lead to the transmission of intestinal pathogens to the conjunctiva and the onset of bleb infection (115). Erosion of the conjunctiva and exposure to the stent are the most common direct causes of endophthalmitis associated with XEN gel stent implantation, and prompt surgical management is essential (103, 115). The exposed stent is generally removed, and intensive infection control measures such as vitrectomy, intravitreal injection of antibiotics, bleb revision, subconjunctival antibiotics, and systemic antibiotics are applied (103). The IOP is managed medically and through other filtering surgeries after complete control of the infection. Simple bleb-related infections without stent exposure can be successfully managed conservatively with systemic and topical antibiotics and dexamethasone, without the need to remove the XEN gel stent. A good prognosis is possible for patients who receive prompt and intensive management, as evidenced by two reported cases that recovered within two lines of vision loss compared to their previous visual acuity (114). To reduce the risk of stent exposure and bleb-related complications, it is advised to use an appropriate surgical technique including posterior application of anti-metabolites, minimizing migration of anti-metabolites toward the limbus, superior placement of the stent away from the lid margin and interpalpebral aperture, and early management of bleb erosion (114).

#### Macular edema

ME is a transient and generally benign condition associated with the combined therapy of XEN gel stent implantation and phacoemulsification. It has been reported to have an incidence of 1.5–4.3% (62, 74, 75). In a study that followed 261 eyes receiving XEN gel stent implantation with or without phacoemulsification for an average of 8.5 months, four cases of ME occurred. All of these cases occurred in eyes receiving the combination therapy and resolved spontaneously without further treatment (75). Oddone et al. reported seven cases of ME during a 12-month follow-up of 239 cases (62). The cause is believed to be post-phacoemulsification Irvine-Gass syndrome, but further studies are needed to confirm its cause and prognosis. The cases were self-limiting and did not have an impact on visual acuity or visual field.

#### Hypertrophic bleb

XEN gel stent implantation surgery creates an artificial drainage pathway for the aqueous humor into the subconjunctival space, where it is mainly absorbed by the conjunctival lymphatics. Similar to other filtering surgeries, it results in a local diffuse bleb that covers approximately one-fourth of the circumference. Hypertrophic bleb is a rare and late complication of XEN gel stent implantation surgery, which occurs months to years after the procedure (Figure 1B). It presents as an extensively enlarged bleb that covers large areas and may cause mechanical ectropion. Interestingly, most hypertrophic blebs form and extend toward the nasal conjunctiva. Tracers injected into the subconjunctival space have revealed that the nasal quadrant of the conjunctiva has three times more outflow pathways than the temporal quadrant, which corresponds to the dominant nasal distribution of the conjunctival lymphatic system (118–120). A retrospective cohort study followed 57 eyes with XEN gel stent implantation for 24 months and reported the development of nasal hypertrophic bleb in five eyes (8.8%). These blebs may recur after needle tapping (76). Managing hypertrophic bleb after XEN gel stent implantation is challenging, and the effects of different management methods have only been reported in a few case studies. Conservative therapies include injecting autologous platelet concentrates, blocking the ab-interno stent with viscoelastic material, and sealing the bleb with tissue adhesive. However, there is a potential risk of extensive bleb adhesion and increased IOP (77, 78). Some surgeons use scleral fixation sutures to restrict the infiltration of aqueous humor into the subconjunctival space and guide its outflow toward the posterior part of the eye (79, 80). A functional bled and drainage pathway is preserved but its efficacy and safety still need further evaluation. Yavuzer and Meşen employed the “drainage channel with sutures” approach to address a hypertrophic bleb complication that arose following the third month of XEN gel implantation (80). Pavičić-Astaloš et al. described a post-operative complication involving dysaesthesia attributed to a large hypertrophic inferonasal bleb that manifested 5 months following XEN implantation. The management involved bleb revision surgery in conjunction with scleral fixation sutures. No post-operative complications were reported, and intraocular pressure (IOP) was effectively controlled during the 20-month follow-up assessment (79).

#### Subconjunctival XEN gel stent fragmentation

The XEN gel stent is a hydrophilic implant made of gelatin, which quickly swells and becomes soft after implantation through hydration (17). Its gelatinous nature makes it highly compatible with the surrounding microenvironment and flexible enough to conform to the curvature within the subconjunctival space. However, there have been reports of breakage and fragmentation of the subconjunctival part when the surgeon attempted to relocate the stent using forceps (105). Novak-Laus et al. also reported a case of “spontaneous” fragmentation of the subconjunctival part of the XEN gel stent discovered during a regular follow-up visit 3 months after surgery in a patient who denied rubbing their eye or experiencing any incidental trauma that could explain the breakage (81). Despite the fragmentation of the stent, it was not replaced because the distal end remained in the Schlemm’s canal and the patient maintained normal IOP (81). Bustros et al. reported a case where a fragment of the XEN gel implant was inadvertently damaged during the needling procedure, 2 months postoperatively. One month later, the patient’s IOP remained controlled, and the bleb functioned well (82). It is pivotal for the surgeons to be cautious during the needling procedure. Particularly in cases where SCH impairs visibility, it is essential to postpone the procedure until optimal visibility can be ensured.

Although breakage of the XEN gel stent is a rare complication, with only two reported cases at present, further testing is required to assess the mechanical strength of the stent. It is important to avoid any forceful grasping of the stent during surgery.

### Late postoperative period (from 6 months)

#### Late spontaneous dislocation of stent

As discussed above, most cases of XEN gel stent dislocation occur relatively soon after surgery due to inappropriate positioning of the stent and erosion of the conjunctiva covering. Late spontaneous dislocation of the XEN gel stent is generally rare and has only been reported in case studies, and its cause remains largely unknown. Boese et al. described a case of a 73-year-old male patient with advanced primary open angle glaucoma who underwent an uncomplicated combined phacoemulsification procedure with *ab interno* gelatin stent implantation. The stent remained in place during the 6-month follow-up period but spontaneously dislocated during a regular follow-up visit without any triggering events or subjective symptoms. The patient denied the history of any trauma or eye rubbing at any point. The cause of spontaneous stent dislocation remained poorly understood, and the authors suspected that insufficient scleral support may lead to the dislocation of the gelatin implant. Another possibility was that repeated deployment during the surgery might have resulted in a looser fit (83). Since further investigation and evidence are lacking, more research is required to calculate the incidence of this rare complication and explore ways to reduce the risk of spontaneous dislocation. Surgeons should maintain close follow-up to promptly detect stent dislocation.

#### Intraocular degradation

The XEN gel stent is made of porcine gelatin crosslinked with glutaraldehyde, which is hydrophilic in nature and quite stable when implanted. The purpose of the crosslinking process is to ensure that the XEN gel stent serves as a permanent device for controlling IOP (16, 121). Preclinical studies have demonstrated that the structure of the XEN gel stent remains intact after 12 months of implantation in dog eyes and over 6 months in nonhuman primate eyes (16). However, a case with the degradation of the XEN gel stent was reported by Widder et al. in a 63-year-old patient, 3 years after implantation. No unique characteristics were identified in this patient, and the degradation primarily affected the intracameral and intrascleral parts of the stent. The degradation caused irregularities in the surface and lumen of the stent, resulting in loss of function. Surgical intervention was required to remove the degraded stent (84). Currently, there has been a lack of long-term observation regarding the implantation of XEN gel stents, and no other reports of intraocular degradation have been documented. However, it is possible that the incidence of intraocular degradation is underestimated, as non-functional stents are typically managed conservatively through needling, and only a few removed stents are carefully examined. Furthermore, it remains unclear whether the degraded materials in the eye are toxic or contribute to further blockage of the aqueous humor drainage system, necessitating further investigation.

## Summary and conclusion

Currently, the implantation of XEN gel stents has been demonstrated as an effective method for controlling IOP in patients with early, moderate, advanced, or refractory glaucoma. Long-term observational studies with follow-up periods of up to 5 years support its safety and efficacy (122). Early postoperative complications of XEN gel stent implantation include HM (1.9–4.6%), occlusion (3.9–8.8%), SCH, CD (0–15%), conjunctival erosion, and exposure of the XEN gel stent (1.1–2.3%). Additionally, there may be incidents of wound and bleb leaks (2.1%) and MG (2.2% incidence). Mid-postoperative complications of XEN gel stent implantation include migration of the XEN stent (1.5% incidence), ptosis (1.2% incidence), endophthalmitis (0.4–3%), ME (1.5–4.3%), hypertrophic bleb (8.8% in 5 out of 57 eyes), and subconjunctival fragmentation (as reported in 2 cases) of the XEN gel stent. Late postoperative complications, which have only been reported in isolated cases, include late spontaneous dislocation and intraocular degradation. Our systematic review was the first comprehensive summary of complications associated with XEN gel stent implantation. It demonstrated rare complications, their incidence, mechanisms, and management methods. Most of these complications are mild and transient, and conservative therapy is usually sufficient. However, when conservative methods fail, surgical management has been shown to be effective. Among these complications, SCH, endophthalmitis, and MG are potentially sight-threatening but rare occurrences in XEN gel stent implantation. Surgeons must pay special attention to these complications. SCH, although rare, can be potentially sight-threatening. Conservative management and early surgical intervention, either through choroidal tap or pars plana vitrectomy, have also been reported (62). Endophthalmitis is a rare but potentially sight-threatening complication. In such cases, the exposed stent is typically removed, and intensive infection control measures such as vitrectomy, intravitreal injection of antibiotics, and systemic antibiotic use are applied (53). As for the management of MG, only one case report discussed the approach. It involves disrupting the anterior displacement of the iris-lens diaphragm, either through laser capsulotomy, hyaloidotomy, iridotomy, or surgical methods like vitrectomy or posterior capsulotomy (69).

The XEN 45 gel stent provides a surgical treatment option for glaucoma that is minimally invasive, resulting in shorter surgical time and less intraoperative discomfort for the patient compared to trabeculectomy. It can be performed as a standalone procedure or combined with phacoemulsification. Although it belongs to the category of MIGS and offers advantages such as reduced tissue damage and quicker surgical time, there is still a risk of severe complications, including endophthalmitis, SCH, and MG. Therefore, close monitoring and early identification of severe complications are crucial for surgeons.

## Author contributions

LG: Data curation, Formal analysis, Investigation, Writing – original draft, Writing – review & editing. LW: Data curation, Formal analysis, Writing – review & editing. JC: Conceptualization, Investigation, Writing – review & editing. LT: Funding acquisition, Writing – review & editing.

## References

[1] Weinreb RN Aung T Medeiros FA. The pathophysiology and treatment of glaucoma: a review. JAMA. (2014) 311:1901–11. doi: 10.1001/jama.2014.3192, PMID: 24825645 PMC4523637

[2] Tham YC Li X Wong TY Quigley HA Aung T Cheng CY. Global prevalence of glaucoma and projections of glaucoma burden through 2040: a systematic review and meta-analysis. Ophthalmology. (2014) 121:2081–90. doi: 10.1016/j.ophtha.2014.05.013, PMID: 24974815

[3] Coleman AL Miglior S. Risk factors for Glaucoma onset and progression. Surv Ophthalmol. (2008) 53:S3–S10. doi: 10.1016/j.survophthal.2008.08.006 19038621

[4] Lichter PR Musch DC Gillespie BW Guire KE Janz NK Wren PA. Interim clinical outcomes in the collaborative initial glaucoma treatment study comparing initial treatment randomized to medications or surgery. Ophthalmology. (2001) 108:1943–53. doi: 10.1016/S0161-6420(01)00873-9 11713061

[5] Coleman AL. Advances in glaucoma treatment and management: surgery. Invest Ophthalmol Vis Sci. (2012) 53:2491–4. doi: 10.1167/iovs.12-9483l 22562849

[6] Koike KJ Chang PT. Trabeculectomy: a brief history and review of current trends. Int Ophthalmol Clin. (2018) 58:117–33. doi: 10.1097/IIO.0000000000000231, PMID: 29870414

[7] De Gregorio A Pedrotti E Stevan G Bertoncello A Morselli S. XEN glaucoma treatment system in the management of refractory glaucomas: a short review on trial data and potential role in clinical practice. Clin Ophthalmol. (2018) 12:773–82. doi: 10.2147/OPTH.S146919, PMID: 29750009 PMC5933334

[8] Hohberger B Welge-Lüßen UC Lämmer R. MIGS: therapeutic success of combined Xen gel stent implantation with cataract surgery. Graefes Arch Clin Exp Ophthalmol. (2018) 256:621–5. doi: 10.1007/s00417-017-3895-3 29335776

[9] De Gregorio A Pedrotti E Russo L Morselli S. Minimally invasive combined glaucoma and cataract surgery: clinical results of the smallest *ab interno* gel stent. Int Ophthalmol. (2018) 38:1129–34. doi: 10.1007/s10792-017-0571-x, PMID: 28555256

[10] Mansouri K Guidotti J Rao HL Ouabas A D’Alessandro E Roy S. Prospective evaluation of standalone XEN gel implant and combined phacoemulsification-XEN gel implant surgery: 1-year results. J Glaucoma. (2018) 27:140–7. doi: 10.1097/IJG.0000000000000858, PMID: 29271806

[11] Asanad S Kalarn S Kaleem MA. Postoperative complications of ab-interno XEN implantation in primary angle closure glaucoma. Am J Clin Exp Immunol. (2021) 10:44–7. PMID: 33815963 PMC8012303

[12] Sng CCA Wang J Hau S Htoon HM Barton K. XEN-45 collagen implant for the treatment of uveitic glaucoma. Clin Exp Ophthalmol. (2018) 46:339–45. doi: 10.1111/ceo.13087, PMID: 29053204

[13] Tailor R Lalias T. A case of refractory neovascular glaucoma treated with a XEN 45 implant. J Glaucoma. (2018) 27:929–30. doi: 10.1097/IJG.0000000000001033, PMID: 30059408

[14] Lin MM Morgan WH Kolomeyer NN Moster SJ Zheng CX Giubilato A. XEN gel stent to treat ICE syndrome: 4 cases. J Glaucoma. (2019) 28:1090–4. doi: 10.1097/IJG.0000000000001341, PMID: 31425336 PMC6888868

[15] Sousa DC Leal I Abegão PL. Steroid-induced protracted severe ocular hypertension in a 14-year-old girl. BMJ Case Rep. (2018) 2018:bcr-2018-225244. doi: 10.1136/bcr-2018-225244, PMID: 29950368 PMC6020893

[16] Lewis RA. *Ab interno* approach to the subconjunctival space using a collagen glaucoma stent. J Cataract Refract Surg. (2014) 40:1301–6. doi: 10.1016/j.jcrs.2014.01.032, PMID: 24943904

[17] Grover DS Flynn WJ Bashford KP Lewis RA Duh YJ Nangia RS. Performance and safety of a new *ab interno* gelatin stent in refractory Glaucoma at 12 months. Am J Ophthalmol. (2017) 183:25–36. doi: 10.1016/j.ajo.2017.07.023 28784554

[18] Kosior-Jarecka E Wróbel-Dudzińska D Święch A Żarnowski T. Bleb compressive sutures in the management of hypotony maculopathy after glaucoma surgery. J Clin Med. (2021) 10:2223. doi: 10.3390/jcm10112223 34063810 PMC8196590

[19] Sheybani A Reitsamer H Ahmed IIK. Fluid dynamics of a novel Micro-fistula implant for the surgical treatment of Glaucoma. Invest Opthalmol Vis Sci. (2015) 56:4789. doi: 10.1167/iovs.15-16625 26218906

[20] Fea AM Durr GM Marolo P Malinverni L Economou MA Ahmed I. XEN(®) gel stent: a comprehensive review on its use as a treatment option for refractory Glaucoma. Clin Ophthalmol. (2020) 14:1805–32. doi: 10.2147/OPTH.S178348, PMID: 32636610 PMC7335291

[21] Buffault J Baudouin C Labbé A. XEN® gel stent for management of chronic open angle glaucoma: a review of the literature. J Franc D’ophtalmol. (2019) 42:e37–46. doi: 10.1016/j.jfo.2018.12.002, PMID: 30683533

[22] Schlenker MB Gulamhusein H Conrad-Hengerer I Somers A Lenzhofer M Stalmans I. Efficacy, safety, and risk factors for failure of standalone *ab interno* gelatin microstent implantation versus standalone trabeculectomy. Ophthalmology. (2017) 124:1579–88. doi: 10.1016/j.ophtha.2017.05.004, PMID: 28601250

[23] Olgun A Duzgun E Yildiz AM Atmaca F Yildiz AA Sendul SY. XEN gel stent versus trabeculectomy: short-term effects on corneal endothelial cells. Eur J Ophthalmol. (2021) 31:346–53. doi: 10.1177/1120672120924339, PMID: 32452237

[24] Zakaria S Ahmed C Mehran NA Sinha S Razeghinejad R Myers JS. Long-term outcomes of subconjunctival gel stent with and without concomitant cataract surgery. Investig Ophthalmol Vis Sci. (2020) 61:947

[25] Vera V Sheybani A Lindfield D Stalmans I Ahmed IIK. Recommendations for the management of elevated intraocular pressure due to bleb fibrosis after XEN gel stent implantation. Clin Ophthalmol. (2019) 13:685–94. doi: 10.2147/OPTH.S195457 31114145 PMC6481982

[26] Moher D Liberati A Tetzlaff J Altman DG. Preferred reporting items for systematic reviews and meta-analyses: the PRISMA statement. Ann Intern Med. (2009) 151:264–9. doi: 10.7326/0003-4819-151-4-200908180-00135 19622511

[27] Karimi A Lindfield D Turnbull A Dimitriou C Bhatia B Radwan M. A multi-centre interventional case series of 259 ab-interno Xen gel implants for glaucoma, with and without combined cataract surgery. Eye. (2018) 33:469–77. doi: 10.1038/s41433-018-0243-8, PMID: 30356133 PMC6460711

[28] Busch T Skiljic D Rudolph T Bergström A Zetterberg M. Four-year outcome of XEN 45 gel stent implantation in a Swedish population. Clin Ophthalmol. (2023) 17:1897–910. doi: 10.2147/OPTH.S412400, PMID: 37425030 PMC10328829

[29] Ferreira NP Pinto LA Marques-Neves C. XEN gel stent internal ostium occlusion: ab-Interno revision. J Glaucoma. (2017) 26:e150–2. doi: 10.1097/IJG.0000000000000625, PMID: 28098579

[30] Tadrosse AF Khouri AS. Laser iridoplasty to treat Iris-occluded XEN gel stent. J Glaucoma. (2020) 29:e91–2. doi: 10.1097/IJG.0000000000001589, PMID: 32568813

[31] Rho S Lim SH. Combined argon laser peripheral iridoplasty and Nd: YAG laser shock wave therapy for recurrent XEN gel stent obstruction due to iris incarceration: a case report. Medicine. (2021) 100:E26652. doi: 10.1097/MD.0000000000026652 34398023 PMC8294893

[32] Scantling-Birch Y Merzougui W Lindfield D. Early postoperative lumen blockage of ab-interno gel stent (XEN) cleared with Nd:YAG laser. Indian J Ophthalmol. (2020) 68:524. doi: 10.4103/ijo.IJO_1051_19, PMID: 32057021 PMC7043161

[33] Zalta AH Sweeney CP Zalta AK Kaufman AH. Intracameral tissue plasminogen activator use in a large series of eyes with valved glaucoma drainage implants. Arch Ophthalmol. (2002) 120:1487–93. doi: 10.1001/archopht.120.11.1487, PMID: 12427061

[34] Zhang Y Xiang H Zhang Y Tang L. Recanalization of Xen45 gel stent implant occlusion using 10 - 0 nylon suture in refractory glaucoma: a case report. BMC Ophthalmol. (2023) 23:418. doi: 10.1186/s12886-023-03109-7, PMID: 37858210 PMC10585744

[35] Bitrian E Song BJ Caprioli J. Bleb revision for resolution of hypotony maculopathy following primary trabeculectomy. Am J Ophthalmol. (2014) 158:597–604.e1. doi: 10.1016/j.ajo.2014.05.021, PMID: 24874999 PMC4314713

[36] Saeedi OJ Jefferys JL Solus JF Jampel HD Quigley HA. Risk factors for adverse consequences of low intraocular pressure after trabeculectomy. J Glaucoma. (2014) 23:e60–8. doi: 10.1097/IJG.0000000000000008, PMID: 24145291

[37] Stamper R. Bilateral chronic hypotony following trabeculectomy with mitomycin-C. J Glaucoma. (2001) 10:325–8. doi: 10.1097/00061198-200108000-00013, PMID: 11558818

[38] Silva RA Doshi A Law SK Singh K. Postfiltration hypotony maculopathy in young Chinese myopic women with glaucomatous appearing optic neuropathy. J Glaucoma. (2010) 19:105–10. doi: 10.1097/IJG.0b013e3181a98a39, PMID: 19661828

[39] Sacchi M Fea AM Monsellato G Tagliabue E Villani E Ranno S. Safety and efficacy of *ab interno* XEN 45 gel stent in patients with Glaucoma and high myopia. J Clin Med. (2023) 12:2477. doi: 10.3390/jcm12072477, PMID: 37048569 PMC10095138

[40] Matsuo H Tomidokoro A Tomita G Araie M. Topical application of autologous serum for the treatment of late-onset aqueous oozing or point-leak through filtering bleb. Eye. (2005) 19:23–8. doi: 10.1038/sj.eye.6701422, PMID: 15254494

[41] Smith MF Magauran RG Betchkal J Doyle JW. Treatment of postfiltration bleb leaks with autologous blood. Ophthalmology. (1995) 102:868–71. doi: 10.1016/S0161-6420(95)30941-4, PMID: 7777292

[42] Higashide T Tagawa S Sugiyama K. Intraoperative Healon5 injection into blebs for small conjunctival breaks created during trabeculectomy. J Cataract Refract Surg. (2005) 31:1279–82. doi: 10.1016/j.jcrs.2004.11.047, PMID: 16105594

[43] Hosoda S Yuki K Ono T Tsubota K. Ophthalmic viscoelastic device injection for the treatment of flat anterior chamber after trabeculectomy: a case series study. Clin Ophthalmol. (2013) 7:1781–5. doi: 10.2147/OPTH.S51165 24043927 PMC3772763

[44] Kurtz S Leibovitch I. Combined perfluoropropane gas and viscoelastic material injection for anterior chamber reformation following trabeculectomy. Br J Ophthalmol. (2002) 86:1225–7. doi: 10.1136/bjo.86.11.1225, PMID: 12386073 PMC1771349

[45] Eha J Hoffmann EM Wahl J Pfeiffer N. Flap suture--a simple technique for the revision of hypotony maculopathy following trabeculectomy with mitomycin C. Graef Arch Clin Exp Ophthalmol. (2008) 246:869–74. doi: 10.1007/s00417-007-0694-2, PMID: 18389274

[46] Shirato S Maruyama K Haneda M. Resuturing the scleral flap through conjunctiva for treatment of excess filtration. Am J Ophthalmol. (2004) 137:173–4. doi: 10.1016/j.ajo.2003.06.001, PMID: 14700662

[47] Haynes WL Alward WL. Rapid visual recovery and long-term intraocular pressure control after donor scleral patch grafting for trabeculectomy-induced hypotony maculopathy. J Glaucoma. (1995) 4:200–1. PMID: 19920669

[48] Vaziri K Schwartz SG Kishor KS Fortun JA Moshfeghi DM Moshfeghi AA. Incidence of postoperative suprachoroidal hemorrhage after glaucoma filtration surgeries in the United States. Clin Ophthalmol. (2015) 9:579–84. doi: 10.2147/OPTH.S78359, PMID: 25897196 PMC4396511

[49] Wang K Wang JC Sarrafpour S. Suprachoroidal hemorrhage after XEN gel implant requiring surgical drainage. J Curr Glauc Pract. (2022) 16:132–5. doi: 10.5005/jp-journals-10078-1378, PMID: 36128082 PMC9452709

[50] Liu JC Green W Sheybani A Lind JT. Intraoperative suprachoroidal hemorrhage during Xen gel stent implantation. Am J Ophthalmol Case Rep. (2020) 17:100600. doi: 10.1016/j.ajoc.2020.100600 32025590 PMC6997831

[51] Prokosch-Willing V Vossmerbaeumer U Hoffmann E Pfeiffer N. Suprachoroidal bleeding after XEN gel implantation. J Glaucoma. (2017) 26:e261–3. doi: 10.1097/IJG.0000000000000795, PMID: 28984715

[52] Rooney DM Shadid HR Siegel LI Watnick RL Lesser GR Obertynski T. Postoperative complications of *ab interno* gelatin microstent. J Glaucoma. (2019) 28:E77–81. doi: 10.1097/IJG.0000000000001194, PMID: 30676413

[53] Smith M Charles R Abdel-Hay A Shah B Byles D Lim LA. 1-year outcomes of the Xen45 glaucoma implant. Eye. (2019) 33:761–6. doi: 10.1038/s41433-018-0310-1, PMID: 30552422 PMC6707157

[54] Gillmann K Bravetti GE Rao HL Mermoud A Mansouri K. Combined and stand-alone XEN 45 gel stent implantation: 3-year outcomes and success predictors. Acta Ophthalmol. (2021) 99:e531–9. doi: 10.1111/aos.14605 32930515

[55] Reitsamer H Sng C Vera V Lenzhofer M Barton K Stalmans I. Two-year results of a multicenter study of the *ab interno* gelatin implant in medically uncontrolled primary open-angle glaucoma. Graef Arch Clin Exp Ophthalmol. (2019) 257:983–96. doi: 10.1007/s00417-019-04251-z, PMID: 30758653

[56] Cutolo CA Negri L Olivari S Cappelli F Traverso CE Iester M. Choroidal detachment after XEN gel stent implantation. J Ophthalmol. (2021) 2021:1–5. doi: 10.1155/2021/6674505 PMC7960055 33747555

[57] Arnould L Theillac V Moran S Gatinel D Grise-Dulac A. Recurrent exposure of XEN gel stent implant and conjunctival Erosion. J Glaucoma. (2019) 28:e37–40. doi: 10.1097/IJG.0000000000001146, PMID: 30676470

[58] Kingston EJ Zagora SL Symes RJ Raman P McCluskey PJ Lusthaus JA. Infective necrotizing Scleritis after XEN gel stent with Mitomycin-C. J Glaucoma. (2022) 31:129–32. doi: 10.1097/IJG.0000000000001959, PMID: 34731869

[59] Santamaria-Alvarez JF Lillo-Sopena J Sanz-Moreno S Caminal-Mitjana JM. Management of conjunctival perforation and XEN gel stent exposure by stent repositioning through the anterior chamber. J Glaucoma. (2019) 28:e24–6. doi: 10.1097/IJG.0000000000001109 30312285

[60] Wu Z Huang C Huang Y Zhang W Ma D. Soft bandage contact lenses in management of early bleb leak following trabeculectomy. Eye Sci. (2015) 30:13–7. doi: 10.1016/s0161-6420(01)00763-1 PMID: 26390792

[61] Aktas Z Aribas YK Bilgihan K Tefon AB. Collagen crosslinking-assisted treatment of a bleb leak: enhancement of vascularization around the bleb. J Curr Glauc Pract. (2021) 15:36–9. doi: 10.5005/jp-journals-10078-1290, PMID: 34393455 PMC8322592

[62] Oddone F Roberti G Giammaria S Posarelli C Ghirelli G Mastropasqua L. Effectiveness and safety of XEN45 implant over 12 months of follow-up: data from the XEN-Glaucoma treatment registry. Eye. (2024) 38:103–11. doi: 10.1038/s41433-023-02642-5, PMID: 37414935 PMC10764778

[63] Bochmann F Azuara-Blanco A. Interventions for late trabeculectomy bleb leak. Cochrane Database Syst Rev. (2012) CD006769. doi: 10.1002/14651858.CD006769.pub2, PMID: 22972097 PMC11663501

[64] Lázaro-Rodríguez V Casado-López D Tolosa FR. Conjunctival collagen crosslinking for the management of bleb leak. Indian J Ophthalmol. (2023) 71:276–9. doi: 10.4103/ijo.IJO_1444_22 36588250 PMC10155536

[65] Chan HMH Choy BNK Lai JSM. Effects of riboflavin and ultraviolet illumination on the biomechanical properties of conjunctiva. Ophthalmic Res. (2018) 60:87–93. doi: 10.1159/000478051, PMID: 28813714

[66] Schlenker MB Ong JA Wu P Jinapriya D Zack B Dorey MW. Surgeon experience as a risk factor for short-term failure for *ab interno* gelatin microstent: a Canadian multicenter propensity-matched study. Ophthalmol Glaucoma. (2022) 5:67–76. doi: 10.1016/j.ogla.2021.05.009, PMID: 34089949

[67] Luntz MH Rosenblatt M. Malignant glaucoma. Surv Ophthalmol. (1987) 32:73–93. doi: 10.1016/0039-6257(87)90101-9 3317956

[68] Montolío Marzo S Lanzagorta Aresti A Davó Cabrera JM Alfonso Muñóz EA Piá Ludeña JV Palacios PE. Malignant glaucoma after XEN45 implant. Arch Soc Esp Oftalmol. (2019) 94:134–7. doi: 10.1016/j.oftal.2018.10.023, PMID: 30578069

[69] Fekih O Zgolli HM Mabrouk S Abdejelil A Zeghal I Nacef L. Malignant glaucoma management: literature review. Tunis Med. (2019) 97:945–9. doi: 10.1038/s41433-018-0243-8 PMID: 32173840

[70] Dervenis N Mikropoulou AM Dervenis P Lewis A. Dislocation of a previously successful XEN glaucoma implant into the anterior chamber: a case report. BMC Ophthalmol. (2017) 17:148. doi: 10.1186/s12886-017-0540-1, PMID: 28830369 PMC5568059

[71] Heidinger A Schwab C Lindner E Riedl R Mossböck G. A retrospective study of 199 Xen45 stent implantations from 2014 to 2016. J Glaucoma. (2019) 28:75–9. doi: 10.1097/IJG.0000000000001122, PMID: 30461555

[72] Linton E Au L. Technique of Xen implant revision surgery and the surgical outcomes: a retrospective interventional case series. Ophthalmol Therapy. (2020) 9:149–57. doi: 10.1007/s40123-020-00234-0, PMID: 32062789 PMC7054468

[73] Ibáñez-Muñoz A Soto-Biforcos VS Rodríguez-Vicente L Ortega-Renedo I Chacón-González M Rúa-Galisteo O. XEN implant in primary and secondary open-angle glaucoma: a 12-month retrospective study. Eur J Ophthalmol. (2020) 30:1034–41. doi: 10.1177/1120672119845226, PMID: 31018685

[74] Szigiato AA Touma S Jabbour S Lord F Agoumi Y Singh H. Efficacy of ab-interno gelatin microstent implantation in primary and refractory glaucoma. Can J Ophthalmol. (2023) 58:328–37. doi: 10.1016/j.jcjo.2022.02.012, PMID: 35339436

[75] Widder RA Dietlein TS Dinslage S Kühnrich P Rennings C Rössler G. The XEN45 gel stent as a minimally invasive procedure in glaucoma surgery: success rates, risk profile, and rates of re-surgery after 261 surgeries. Graef Arch Clin Exp Ophthalmol. (2018) 256:765–71. doi: 10.1007/s00417-018-3899-7, PMID: 29356886

[76] Wanichwecharungruang B Ratprasatporn N. 24-month outcomes of XEN45 gel implant versus trabeculectomy in primary glaucoma. PLoS One. (2021) 16:e0256362. doi: 10.1371/journal.pone.0256362, PMID: 34411152 PMC8376039

[77] Jonas JB Dugrillon A Klüter H Kamppeter B. Subconjunctival injection of autologous platelet concentrate in the treatment of overfiltrating bleb. J Glaucoma. (2003) 12:57–8. doi: 10.1097/00061198-200302000-00012, PMID: 12567114

[78] Fernández-García A Romero C Garzón N. “Dry Lake” technique for the treatment of hypertrophic bleb following XEN(®) gel stent placement. Arch Soc Esp Oftalmol. (2015) 90:536–8. doi: 10.1016/j.oftal.2015.03.003 26008922

[79] Pavicic-Astalos J Ankamah E Nolan JM Ng E Garcia-Feijoo J. The use of fixation suture to treat Inferonasal hypertrophic bleb after Xen gel stent implant: a case report. Case Rep Ophthalmol. (2022) 13:253–8. doi: 10.1159/000523906, PMID: 35611011 PMC9082189

[80] Yavuzer K Mesen A. The treatment of a hypertrophic bleb after XEN gel implantation with the “Drainage Channel with sutures” method: a case report. BMC Ophthalmol. (2019) 19:245. doi: 10.1186/s12886-019-1249-0, PMID: 31795968 PMC6892140

[81] Novak-Laus K Knezevic L Maric G Zoric Geber M Vatavuk Z. Subconjunctival fragmentation of a previously efficient Xen gel stent implantation and successful bleb formation: a case report. Acta Clin Croat. (2019) 58:767–70. doi: 10.20471/acc.2019.58.04.25, PMID: 32595262 PMC7314297

[82] Bustros Y Chaudhary A Salinas L Mansouri K. Cutting the subconjunctival fragment of the XEN gel implant during needling procedure. Eur J Ophthalmol. (2020) 30:NP11–5. doi: 10.1177/1120672118805876 30328376

[83] Boese EA Shah M. Late spontaneous dislocation of an *ab interno* gelatin microstent. J Glaucoma. (2018) 27:e84–6. doi: 10.1097/IJG.0000000000000897, PMID: 29401159

[84] Widder RA Kuhnrich P Hild M Rennings C Szumniak A Rossler GF. Intraocular degradation of XEN45 gel stent 3 years after its implantation. J Glaucoma. (2019) 28:e171–3. doi: 10.1097/IJG.0000000000001364, PMID: 31517762

[85] Gillmann K Mansouri K Bravetti GE Mermoud A. Chronic intraocular inflammation as a risk factor for XEN gel stent occlusion: a case of microscopic examination of a fibrin-obstructed XEN stent. J Glaucoma. (2018) 27:739–41. doi: 10.1097/IJG.0000000000001002, PMID: 29877971

[86] Eagle RC Razeghinejad R. Xen gel stent occlusion with iris pigment epithelium. Clin Experiment Ophthalmol. (2020) 48:258–9. doi: 10.1111/ceo.13658, PMID: 31613037

[87] Gillmann K Bravetti GE Mansouri K. Delayed obstruction of XEN gel stent by cell debris in primary open-angle Glaucoma: a new insight into the pathophysiology of filtration device failure. J Curr Glaucoma Pract. (2019) 13:113–5. doi: 10.5005/jp-journals-10078-1258, PMID: 32431478 PMC7221242

[88] Oyakhire JO Moroi SE. Clinical and anatomical reversal of long-term hypotony maculopathy. Am J Ophthalmol. (2004) 137:953–5. doi: 10.1016/j.ajo.2003.11.019, PMID: 15126172

[89] Harizman N Ben-Cnaan R Goldenfeld M Levkovitch-Verbin H Melamed S. Donor scleral patch for treating hypotony due to leaking and/or overfiltering blebs. J Glaucoma. (2005) 14:492–6. doi: 10.1097/01.ijg.0000185618.98915.d2 16276283

[90] Bochmann F Kaufmann C Kipfer A Thiel MA. Corneal patch graft for the repair of late-onset hypotony or filtering bleb leak after trabeculectomy: a new surgical technique. J Glaucoma. (2014) 23:e76–80. doi: 10.1097/IJG.0000000000000014, PMID: 24145292

[91] Panday M Shantha B George R Boda S Vijaya L. Outcomes of bleb excision with free autologous conjunctival patch grafting for bleb leak and hypotony after glaucoma filtering surgery. J Glaucoma. (2011) 20:392–7. doi: 10.1097/IJG.0b013e3181e87efc, PMID: 20616750

[92] Dietlein TS Lappas A Rosentreter A. Secondary subconjunctival implantation of a biodegradable collagen-glycosaminoglycan matrix to treat ocular hypotony following trabeculectomy with mitomycin C. Br J Ophthalmol. (2013) 97:985–8. doi: 10.1136/bjophthalmol-2013-303357, PMID: 23759438

[93] Tuli SS WuDunn D Ciulla TA Cantor LB. Delayed suprachoroidal hemorrhage after glaucoma filtration procedures. Ophthalmology. (2001) 108:1808–11. doi: 10.1016/S0161-6420(01)00763-1, PMID: 11581053

[94] Jeganathan VS Ghosh S Ruddle JB Gupta V Coote MA Crowston JG. Risk factors for delayed suprachoroidal haemorrhage following glaucoma surgery. Br J Ophthalmol. (2008) 92:1393–6. doi: 10.1136/bjo.2008.141689, PMID: 18684750

[95] Nadarajah S Kon C Rassam S. Early controlled drainage of massive suprachoroidal hemorrhage with the aid of an expanding gas bubble and risk factors. Retina. (2012) 32:543–8. doi: 10.1097/IAE.0b013e31822058e9, PMID: 21955989

[96] Healey PR Herndon L Smiddy W. Management of suprachoroidal hemorrhage. J Glaucoma. (2007) 16:577–9. doi: 10.1097/IJG.0b013e318156a5a9 17873722

[97] Haga A Inatani M Shobayashi K Kojima S Inoue T Tanihara H. Risk factors for choroidal detachment after trabeculectomy with mitomycin C. Clin Ophthalmol. (2013) 7:1417–21. doi: 10.2147/OPTH.S46375, PMID: 23874083 PMC3713998

[98] Iwasaki K Kakimoto H Arimura S Takamura Y Inatani M. Prospective cohort study of risk factors for choroidal detachment after trabeculectomy. Int Ophthalmol. (2020) 40:1077–83. doi: 10.1007/s10792-019-01267-6, PMID: 31989350

[99] Jampel HD Musch DC Gillespie BW Lichter PR Wright MM Guire KE. Perioperative complications of trabeculectomy in the collaborative initial glaucoma treatment study (CIGTS). Am J Ophthalmol. (2005) 140:16–22. doi: 10.1016/j.ajo.2005.02.013, PMID: 15939389

[100] Pérez-Torregrosa VT Olate-Pérez Á Cerdà-Ibáñez M Gargallo-Benedicto A Osorio-Alayo V Barreiro-Rego A. Combined phacoemulsification and XEN45 surgery from a temporal approach and 2 incisions. Arch Soc Esp Oftalmol. (2016) 91:415–21. doi: 10.1016/j.oftal.2016.02.006, PMID: 26995503

[101] Galal A Bilgic A Eltanamly R Osman A. XEN Glaucoma implant with Mitomycin C 1-year follow-up: result and complications. J Ophthalmol. (2017) 2017:1–5. doi: 10.1155/2017/5457246 PMC5350531 28348884

[102] Hengerer FH Kohnen T Mueller M Conrad-Hengerer I. *Ab interno* gel implant for the treatment of Glaucoma patients with or without prior Glaucoma surgery: 1-year results. J Glaucoma. (2017) 26:1130–6. doi: 10.1097/IJG.0000000000000803 29035911

[103] Lapira M Cronbach N Shaikh A. Extrusion and breakage of XEN gel stent resulting in Endophthalmitis. J Glaucoma. (2018) 27:934–5. doi: 10.1097/IJG.0000000000001058, PMID: 30113510

[104] Wells AP Cordeiro MF Bunce C Khaw PT. Cystic bleb formation and related complications in limbus- versus fornix-based conjunctival flaps in pediatric and young adult trabeculectomy with mitomycin C. Ophthalmology. (2003) 110:2192–7. doi: 10.1016/S0161-6420(03)00800-5, PMID: 14597529

[105] Olate-Pérez Á Pérez-Torregrosa VT Gargallo-Benedicto A Escudero-Igualada R Cerdà-Ibáñez M Barreiro-Rego A. Management of conjunctival perforation and late Seidel after XEN(®) surgery. Arch Soc Esp Oftalmol. (2018) 93:93–6. doi: 10.1016/j.oftal.2017.10.002, PMID: 29224970

[106] Montolio-Marzo S Lanzagorta-Aresti A Pia-Ludena JV Davo-Cabrera JM. Conjunctival bleb tearing by XEN gel stent after conjunctival compression sutures. Eur J Ophthalmol. (2022) 32:NP76–8. doi: 10.1177/1120672120970862 33176452

[107] Salinas L Chaudhary A Guidotti J Mermoud A Mansouri K. Revision of a leaking bleb with XEN gel stent replacement. J Glaucoma. (2018) 27:e11–3. doi: 10.1097/IJG.0000000000000811, PMID: 29088056

[108] Ruben S Tsai J Hitchings RA. Malignant glaucoma and its management. Br J Ophthalmol. (1997) 81:163–7. doi: 10.1136/bjo.81.2.163, PMID: 9059253 PMC1722113

[109] Foreman-Larkin J Netland PA Salim S. Clinical Management of Malignant Glaucoma. J Ophthalmol. (2015) 2015:283707:1–6. doi: 10.1155/2015/283707 26819754 PMC4706935

[110] Rainin EA Carlson BM. Postoperative diplopia and ptosis. A clinical hypothesis based on the myotoxicity of local anesthetics. Arch Ophthalmol. (1985) 103:1337–9. doi: 10.1001/archopht.1985.01050090089038 4038126

[111] Crosby NJ Shepherd D Murray A. Mechanical testing of lid speculae and relationship to postoperative ptosis. Eye. (2013) 27:1098–101. doi: 10.1038/eye.2013.133, PMID: 23788211 PMC3772367

[112] Bernardino CR Rubin PA. Ptosis after cataract surgery. Semin Ophthalmol. (2002) 17:144–8. doi: 10.1076/soph.17.3.144.14782 12759843

[113] Ali ZC Khoo DI Stringa F Shankar V. Migration of xen45 implant: findings, mechanism, and management. J Curr Glauc Pract. (2019) 13:79–81. doi: 10.5005/jp-journals-10078-1253, PMID: 31564799 PMC6743307

[114] Kerr NM Wang J Sandhu A Harasymowycz PJ Barton K. *Ab interno* gel implant-associated bleb-related infection. Am J Ophthalmol. (2018) 189:96–101. doi: 10.1016/j.ajo.2018.02.014, PMID: 29499173

[115] Lim R Lim KS. XEN implant-related endophthalmitis. Ophthalmology. (2018) 125:209. doi: 10.1016/j.ophtha.2017.10.019, PMID: 29389405

[116] Karri B Gupta C Mathews D. Endophthalmitis following XEN stent exposure. J Glaucoma. (2018) 27:931–3. doi: 10.1097/IJG.0000000000001012 29952822

[117] Higginbotham EJ Stevens RK Musch DC Karp KO Lichter PR Bergstrom TJ. Bleb-related endophthalmitis after trabeculectomy with mitomycin C. Ophthalmology. (1996) 103:650–6. doi: 10.1016/S0161-6420(96)30639-8 8618766

[118] Akiyama G Saraswathy S Bogarin T Pan X Barron E Wong TT. Functional, structural, and molecular identification of lymphatic outflow from subconjunctival blebs. Exp Eye Res. (2020) 196:108049. doi: 10.1016/j.exer.2020.108049, PMID: 32387381 PMC7328765

[119] Schulte-Merker S Sabine A Petrova TV. Lymphatic vascular morphogenesis in development, physiology, and disease. J Cell Biol. (2011) 193:607–18. doi: 10.1083/jcb.201012094, PMID: 21576390 PMC3166860

[120] Gong P Yu DY Wang Q Yu PK Karnowski K Heisler M. Label-free volumetric imaging of conjunctival collecting lymphatics ex vivo by optical coherence tomography lymphangiography. J Biophotonics. (2018) 11:e201800070. doi: 10.1002/jbio.201800070, PMID: 29920959

[121] Yang G Xiao Z Long H Ma K Zhang J Ren X. Assessment of the characteristics and biocompatibility of gelatin sponge scaffolds prepared by various crosslinking methods. Sci Rep. (2018) 8:1616. doi: 10.1038/s41598-018-20006-y, PMID: 29371676 PMC5785510

[122] Chaudhary A Salinas L Guidotti J Mermoud A Mansouri K. XEN gel implant: a new surgical approach in glaucoma. Expert Rev Med Devices. (2018) 15:47–59. doi: 10.1080/17434440.2018.1419060, PMID: 29258404

