# Supplementary material for: Complications of XEN gel stent implantation for the treatment of glaucoma: a systematic review
Source: Front Med (Lausanne). 2024 May 6;11:1360051. doi: 10.3389/fmed.2024.1360051 (PMC11102958; doi:10.3389/fmed.2024.1360051)
Supplement: Supplementary file 1 [file Table_1.docx]

**Supplementary material**

**Supplementary Table 1** Information and major outcomes of included studies for the meta-analysis.

| **Author** | **Type of article** | **Number of patients** | **Surgery** | **Type of Glaucoma** | **Follow-up time** | **Complications(N)** | **The time of complications** | **Reason of complication** | **Interventions** |
| --- | --- | --- | --- | --- | --- | --- | --- | --- | --- |
| Zaria C Ali et al(1) | case | 1 | XEN | PACG | 5m | migrated into the anterior chamber | 4m | XEN is positioned such that more than 2 mm  is in the subconjunctival space | explanted a new XEN |
| Samuel Asanad et al(2) | case | 1 | XEN+Phaco | PACG | 2m | occlusion | 2m | iris pigmentary deposits | Ab-interno approach,eplaned  Medication and scheduled for surgical revision of the stent. |
| Nuno P Ferreira et al(3) | case | 1 | XEN | bilateral POAG secondary to pseudoexfoliation | 3m | early bleb failure (thickened, non-diffuse bleb) | 3m | N.A. | Needling of the bleb assisted with vital dye was performed |
| Lim,R.and K.s.Lim(4) | case | 1 | XEN | N.A. | N.A. | endophthalmitis | 11m | An exposes XEN 45 | Intravitreal and topical antibiotics |
| Kevin Gillmann(5) | case | 1 | XEN-augmented Baerveldt | bullous corneal decompensation and intraocular hypertension | 8m | occlusion | 6m | Anterior chamber fibrin formation | stent was removed and replaced by a Baerveldt tube |
| Jasna Pavičić-Astaloš(6) | case | 1 | XEN+PHACO | POAG | 20m | Inferonasal Hypertrophic Bleb | 5m | N.A. | The Use of Fixation Suture |
| Verena Prokosch-Willing et al(7) | case | 1 | XEN | pseudoexfoliation glaucoma | 1.5m | Suprachoroidal bleeding | 2d | Risk factors are high preoperative IOP, abrupt IOP decrease or postoperative hypotony | mannitol infusion and Atropine0.5% eyedrops were  administered 6 times a day |
| Erin A. Boese, MD et al(8) | case | 1 | XEN+Phaco | POAG | 6m | Late Spontaneous Dislocation | 6m | Unclear  repeated doses ofantimetabolites or the new administration of subconjunctial bevacizumab | dislocated gelatin microstent implant was explanted using anterior segment microforceps |
| Bhavani Karri et al(9) | case | 1 | XEN | POAG | 6m | endophthalmitis | 4m | erosion  coinciding gastrointestinal disease and poor handwashing | Removed stent,  pars plana vitrectomy, and intravitreal antibiotics |
| Matthew Lapira et al(10) | case | 1 | XEN | pseudoexfoliative glaucoma | N.A. | endophthalmitis | 4m | Extrusion and Breakage | vitreoretinal service and underwent pars plana vitrectomy and conjunctival exploration |
| Katia Novak-Lauš(11) | case | 1 | XEN | POAG | N.A. | subconjunctival fragmentation | 3m | The mechanism of its occurrence remains unclear. | Neither serious complications nor intraocular pressure increase were detected |
| Juan F. Santamaría-Álvarez(12) | case | 1 | XEN | POAG | 5m | exposure | 3m | continuous friction between the distal end of the stent and the conjunctiva—due to blinking or due to a malpositioned implant—may have caused the bleb leak. | reposition  the stent through the anterior chamber in order to shorten the  subconjunctival path and to suture the conjunctival defect. |
| Yarrow Scantling-Birch(13) | case | 1 | XEN+Phaco | PACG | 2m | fibrin plug in proximal segment of XEN stent | 1d | N.A. | Post YAG laser lumen |
| Filippo Tatti et al(14) | case | 1 | XEN | POAG | 2m | occluded proximal end of the XEN stent | 1m | Vitreoretinal surgery | Ab interno trimming with vitreous scissors |
| Salinas, L., et al.(15) | case | 1 | XEN+Phaco | bilateral pseudoexfoliation glaucoma | 1m | leaking bleb | 2w | N.A. | XEN Gel Stent, managed by implantation of a new XEN Gel Stent and ab-externo bleb revision with removal of the old XEN Gel Stent |
| Olate-Pérez, Á., et al(16) | case | 1 | XEN | POAG | 23m | conjunctival perforation | 18m | Conjunctival coverage on its own (with or without a graft)  wouldhave produced a new perforation due to the short sub-  conjunctival length of the XEN® implant. | dissection, forward displacement and conjunctival suture, covering by means of grafts and/or stent extraction. |
| Louis Arnould et al(17) | case | 1 | XEN | POAG | 3m | recurrent exposure of Stent and conjunctival erosion | 2m | N.A. | use of a free  conjunctival autograf |
| S.Montolío Marzo et al(18) | Case | 1 | XEN+Phaco | POAG | 1m | Malignant glaucoma | 4d | N.A. | complete pars plana vitrectomy  Ahmed valve |
| Kevin Wang(19) | case | 1 | XEN | POAG | 2m | Suprachoroidal Hemorrhage | 3d | hypotony and anticoagulation use. | Surgical Drainage |
| James C. Liu et al(20) | case | 1 | XEN | POAG | 6m | Intraoperative suprachoroidal hemorrhage | during Xen gel stent  implantation | hypotony | Conservative medical management |
| Seungsoo Rho et al(21) | case | 1 | XEN | POAG | 3m | recurrent  XEN gel stent obstruction due to iris incarceration | 10d | the iris and intraluminal pigment dispersion | Combined ALPI and low energy Nd: YAG laser shock wave therapy |
| Randolf A. Widder et al(22) | case | 1 | XEN |  | 2Y | Intraocular Degradation(rarereport) | 2Y | degradation of the XEN45 Gel Stent has  not been described. | removed |
| Kamil Yavuzer et al(23) | case | 1 | XEN+Phaco | pseudoexfoliation glaucoma | N.A. | hypertrophic bleb | 3m | N.A. | Drainage Channel with Sutures |
| Abanoob F et al(24) | case | 1 | XEN | POAG | 6m | Iris-occluded | 10d | It  may thus be important to avoid a steeper “plunge” into the  AC which may point the stent in the direction of the iris.  Avoiding AC shallowing and rubbing the eye is  paramount. | YAG-laser iridotomy |
| Nikolaos Dervenis(25) | case | 1 | XEN | POAG | 6m | dislocation of the XEN  implant into the anterior chamber | 6m | Potentially inflammation of the episclera or external forces  applied by the patient during the inflammation could have  resulted to the implant dislocation | removed |
| A. Fernández-García(26) | case | 1 | XEN | POAG |  | hypertrophic bleb | 2d | N.A. | drain the hypertrophic bleb following  blockage with viscoelastic of the ab-interno stent and bleb sealing with a tissue adhesive. |
| Á. Olate-Pérez(27) | case | 1 | XEN+Phaco | POAG | 18m | conjunctival perforation | 6m | falta de cobertura palpebral, localización super-  ficial del implante y una longitud < 1,5 mm del trayecto  subconjuntival | conjuntiva y suturarla |
| Shu Yu Tan(28) | case | 3 | XEN | refractory steroid-induced glaucoma after corneal lamellar keratoplasty | 12m | hypotony maculopathy, stent  migration and hyphaema | 2d | acute gouty arthritis | repositioned using intraocular forcep |
| Yao Zhang et al(29) | case | 1 | XEN | JOAG | 11m | an anterior chamber tip occlusion | 6d | fibrin plugs or  cellular debris | 10−0 nylon suture to  recanalize Xen45 Gel Stent |
| Emma Linton(30) | Case series | 16 | XEN | 13 POAG  2 Uveitic glaucoma  1 Pseudoexfoliation | 3m | 1 uffered  hypotonous maculopathy  1 bleb-related  Endophthalmitis  not functioning during the  revision procedure | 3m | N.A. | self-resolved without treatment  this patient had a history of nasolacrimal duct  obstruction and recurrent conjunctivitis  converted to a trabeculectomy |
| Chelvin C. A. Sng et al(31) | Case series | 31 | XEN+Phaco | POAG(12)  PACG(19) | 12m | Table3  transient hypotony (12.9%)  ptosis (12.9%)  cystoid macular edema  occlusion with iris(3.2%) | 1m  1w | N.A. | Medication  laser iridoplasty |
| Rooney, D. M., et al.(32) | Case series | 4 | 2XEN  1XEN+Phaco  XEN  +Phaco | POAG | 1m  3w  3m  9m | 1. suprachoroidal hemorrhage and a macula-involving rhegmatogenous retinal detachment 2. subconjunctival portion of the XEN was curled within a low diffuse bleb 3. occluded the stent’ 4. occluded by a portion of Descemet’s membrane | 2d  1m  4w  5w | N.A. | 1.Surgery  XEN remove  2.Reposition  Remove and trabeculectomy with MMC  3. Nd:YAG  laser lysis of the membrane  4.Nd:YAG laser |
| Nathan M. Kerr et al(33) | Case series | 3 | XEN  XEN+ femtosecond laser assisted  cataract surgery  XEN | secondary glaucoma  primary angle closure  secondary glaucoma | N.A. | ate bleb-related infection occurring after ab interno gelatin  stent insertion | 8, 7 and 24 m | that long-term topical steroids required to control inflammation in eyes with uveitic glaucoma may predispose to bleb-related infection | on topical moxifloxacin 0.5%  every 30 minutes and oral moxifloxacin 400 mg daily  topical moxifloxacin 0.5% and steroid as well as systemic moxifloxacin, A core vitrectomy was performed  vitreous tap and  intravitreal injection of vancomycin, amikacin, and dexamethasone |
| Maria E. Galimi et al(34) | retrospective | 170eyes | XEN | POAG | 4w | hypotony | 1w | mostly in myopic eyes |  |
| Randolf A. Widder et al(35) | retrospective | 261(234eyes) | XEN(184)  XEN+Phaco(49) | POAG | 12m | Two stents eroded the conjunctiva  Table 4side-effects | N.A. | N.A. | One patient received a new sten |
| Fritz H. Hengerer(36) | retrospective | 146(242eyes) | XEN | refractory to anti-glaucoma medication or glaucoma  surgery. | 12m | 9eyes hypotony  29eeyes cysts | 1m  3m | N.A. | 2eyes anterior chamber refill with OVD |
| Boonsong Wanichwecharungruang(37) | retrospective | 118eyes | 57XEN | POAG  PACG | 24m | Table3  Ptosis6 eyes  Extrusion 3eyes    Fracture 1eye  Nasal bleb 5eyes | 1m | First, lid speculum could injure the levator aponeurosis. Second, MMC and xylocaine be toxic to it. . | subsequent ptosis correction  Needling revision  Removed  drainage channel with sutures |
| Michael Smith(38) | retrospective | 68eyes | 10 Xen+Phaco  58 Xen alone | Primary open angle/normal tension  glaucoma 59 (86.7%)  Pseudoexfoliative glaucoma 4 (5.9%)  PACG 3  (4.4%)  Ocular hypertension 2 (2.9%) | 12m | 3 overhanging blebs  1 exposure  5 hypotony  1 choroidal effusions | 12m | N.A. | bleb  revision  evision with  pericardial patch graft. |
| SZ Tan(39) | retrospective | 39eyes | 43XEN  4XEN+Phaco | POAG30  Pseudoexfoliation 2  Pigment dispersion syndrome 1  Uveitic 4  Rubeotic 1 Steroid induced 1 | 1Y | 3 obstructed  1 hyphaema  8 numerical  hypotony | 4w | N.A | focal Argon laser  iridoplasty.  requiring AC washout  1 required AC reformation at week 3. |
| Ayesha Karimi(40) | retrospective | 259 | XEN | 208  (80.3%) POAG;  13 (5.0%) PXF, 6 (2.3%)  PDS,  5 (1.9%) NVG | 18m | implant  exposure in 6cases; persistent hypotonous maculopathy in 5 cases; persistent choroidal effusions in 4cases; a cyclodialysis cleft secondary to implant insertion in 1 case; and 1 case of endophthalmitis post-  implant bleb resuturing | 1w  1m | implant exposure  secondary to bleb leak  endophthalmitis following a bleb  leak | required resuturing |
| Matteo Sacchi(41) | retrospective | 14 | 7 XEN | High myopiawith POAG | 2Y | Hypotony2  Choroidal detachment1 | N.A | Myopia | N.A |
| Carlo Alberto Cutolo(42) | retrospective | 126 patients,25 (19.8%) | XEN | POAG  XFG | 1m | choroidal detachment  22(88%) eyes, 3 (12%) eyes haemorrhagic CD | 1m | hypotony and uveal inflammation | N.A |
| Matthew B.Schlenker,Msc(43) | retrospective | 270eyes | XEN | POAG  PXF | 1Y | blocked | 1m | N.A | needling |
| DAVINDER S. GROVER(44) | prospective | 65 | XEN | POAG  PXF | 12m | stent exposure | N.A | N.A | repaired |
| Herbert Reitsamer(45) | prospective | 218eyes | XEN(120)  Phaco + implant(98) | POAG | 2Y | 5 Hypotony  4 Conjunctival erosion  3 Implant blockage by iris  2 Blepharitis  1 Endophthalmitis  1Macular edema | 12m | 1m  15m | Self-resolved  anterior chamber wash, vitrectomy, and intravitreal an-  tibiotics. |
| Ahmed Galal et al(46) | prospective interventional study | 13eyes | XEN(3)  XEN+Phaco | POAG | 12m | 1.choroidal detachment 2 eyes  2.implant extrusion1 eye  3.2eyes underwent trabeculectomy | 1m | 1. early postoperative hypotony 2. previously scarring and thinning of conjunctiva 3. 3.inadequately controlled IOP by topical mediations | 1.systemic steroids and atropine eye drop   1. Repositioning and conjunctival sutures   3.trabeculectomy |
| Chelvin CA Sng FRCSEd(47) | Prospective case series | 24 | XEN | uveitic glaucoma | 12m | Implant exposure 2eyes  One eye  developed blebitis | 3m,4m  9m | Staphylococcus  aureus. | surgical  conjunctival closure.  topical antibiotics |

Abbreviations in the table:

POAG ;primary open-angle glaucoma;XFG ;exfoliative glaucoma; PXF

Pseudoexfoliation glaucoma; PACG: Primary angle closure glaucoma PDS;pigment dispersion syndrome ; NVG neovascular glaucoma;CD; Choroidal detachment;Phaco phacoemulsification;AC:anterior chamber;Y year ;m ; month ; d ; day; JOAG; juvenile open-angle glaucoma

Reference

1. Ali ZC, Khoo DI, Stringa F, Shankar V. Migration of XEN45 Implant: Findings, Mechanism, and Management. J Curr Glaucoma Pract. 2019;13(2):79-81.

2. Asanad S, Kalarn S, Kaleem MA. Postoperative complications of Ab-Interno XEN implantation in primary angle closure glaucoma. Am J Clin Exp Immunol. 2021;10(1):44-7.

3. Ferreira NP, Pinto JM, Teixeira F, Pinto LA. XEN Gel Stent Early Failure-dye-enhanced Ab-externo Revision. J Curr Glaucoma Pract. 2018;12(3):139-41.

4. Lim R, Lim KS. XEN Implant-Related Endophthalmitis. Ophthalmology. 2018;125(2):209.

5. Gillmann K, Mansouri K, Bravetti GE, Mermoud A. Chronic Intraocular Inflammation as a Risk Factor for XEN Gel Stent Occlusion: A Case of Microscopic Examination of a Fibrin-obstructed XEN Stent. J Glaucoma. 2018;27(8):739-41.

6. Pavicic-Astalos J, Ankamah E, Nolan JM, Ng E, Garcia-Feijoo J. The Use of Fixation Suture to Treat Inferonasal Hypertrophic Bleb after Xen Gel Stent Implant: A Case Report. Case Rep Ophthalmol. 2022;13(1):253-8.

7. Prokosch-Willing V, Vossmerbaeumer U, Hoffmann E, Pfeiffer N. Suprachoroidal Bleeding After XEN Gel Implantation. J Glaucoma. 2017;26(12):e261-e3.

8. Boese EA, Shah M. Late Spontaneous Dislocation of an Ab Interno Gelatin Microstent. J Glaucoma. 2018;27(4):e84-e6.

9. Karri B, Gupta C, Mathews D. Endophthalmitis Following XEN Stent Exposure. J Glaucoma. 2018;27(10):931-3.

10. Lapira M, Cronbach N, Shaikh A. Extrusion and Breakage of XEN Gel Stent Resulting in Endophthalmitis. J Glaucoma. 2018;27(10):934-5.

11. Novak-Laus K, Knezevic L, Maric G, Zoric Geber M, Vatavuk Z. Subconjunctival Fragmentation of a Previously Efficient Xen Gel Stent Implantation and Successful Bleb Formation: A Case Report. Acta Clin Croat. 2019;58(4):767-70.

12. Santamaria-Alvarez JF, Lillo-Sopena J, Sanz-Moreno S, Caminal-Mitjana JM. Management of Conjunctival Perforation and XEN Gel Stent Exposure by Stent Repositioning Through the Anterior Chamber. J Glaucoma. 2019;28(2):e24-e6.

13. Scantling-Birch Y, Merzougui W, Lindfield D. Early postoperative lumen blockage of ab-interno gel stent (XEN) cleared with Nd:YAG laser. Indian J Ophthalmol. 2020;68(3):524.

14. Tatti F, Gentile P, Mangoni L, Demarinis G, Napoli P, Fossarello M. Xen45 gel stent ab interno trimming for ostium occlusion: case report. BMC Ophthalmol. 2021;21(1):446.

15. Salinas L, Chaudhary A, Guidotti J, Mermoud A, Mansouri K. Revision of a Leaking Bleb With XEN Gel Stent Replacement. J Glaucoma. 2018;27(1):e11-e3.

16. Olate-Pérez Á, Pérez-Torregrosa VT, Gargallo-Benedicto A, Escudero-Igualada R, Cerdà-Ibáñez M, Barreiro-Rego A, et al. Management of conjunctival perforation and late Seidel after XEN ® surgery. Archivos de la Sociedad Española de Oftalmología (English Edition). 2018;93(2):93-6.

17. Arnould L, Theillac V, Moran S, Gatinel D, Grise-Dulac A. Recurrent Exposure of XEN Gel Stent Implant and Conjunctival Erosion. J Glaucoma. 2019;28(3):e37-e40.

18. Montolío Marzo S, Lanzagorta Aresti A, Davó Cabrera JM, Alfonso Muñóz EA, Piá Ludeña JV, Palacios Pozo E. Malignant glaucoma after XEN45 implant. Archivos de la Sociedad Española de Oftalmología (English Edition). 2019;94(3):134-7.

19. Wang K, Wang JC, Sarrafpour S. Suprachoroidal Hemorrhage after XEN Gel Implant Requiring Surgical Drainage. J Curr Glaucoma Pract. 2022;16(2):132-5.

20. Liu JC, Green W, Sheybani A, Lind JT. Intraoperative suprachoroidal hemorrhage during Xen gel stent implantation. Am J Ophthalmol Case Rep. 2020;17:100600.

21. Rho S, Lim SH. Combined argon laser peripheral iridoplasty and Nd: YAG laser shock wave therapy for recurrent XEN gel stent obstruction due to iris incarceration: A case report. Medicine (Baltimore). 2021;100(29):e26652.

22. Widder RA, Kuhnrich P, Hild M, Rennings C, Szumniak A, Rossler GF. Intraocular Degradation of XEN45 Gel Stent 3 Years After its Implantation. J Glaucoma. 2019;28(12):e171-e3.

23. Yavuzer K, Mesen A. The treatment of a hypertrophic bleb after XEN gel implantation with the "Drainage Channel with sutures" method: a case report. BMC Ophthalmol. 2019;19(1):245.

24. Tadrosse AF, Khouri AS. Laser Iridoplasty to Treat Iris-occluded XEN Gel Stent. J Glaucoma. 2020;29(8):e91-e2.

25. Dervenis N, Mikropoulou AM, Dervenis P, Lewis A. Dislocation of a previously successful XEN glaucoma implant into the anterior chamber: a case report. BMC Ophthalmol. 2017;17(1):148.

26. Fernández-García A, Romero C, Garzón N. "Dry Lake" technique for the treatment of hypertrophic bleb following XEN(®) Gel Stent placement. Arch Soc Esp Oftalmol. 2015;90(11):536-8.

27. Olate-Perez A, Perez-Torregrosa VT, Gargallo-Benedicto A, Escudero-Igualada R, Cerda-Ibanez M, Barreiro-Rego A, et al. Management of conjunctival perforation and late Seidel after XEN((R)) surgery. Arch Soc Esp Oftalmol (Engl Ed). 2018;93(2):93-6.

28. Tan SY, Md Din N, Mohd Khialdin S, Wan Abdul Halim WH, Tang SF. Ab-Externo Implantation of XEN Gel Stent for Refractory Steroid-Induced Glaucoma After Lamellar Keratoplasty. Cureus. 2021;13(2):e13320.

29. Zhang Y, Xiang H, Zhang Y, Tang L. Recanalization of Xen45 gel stent implant occlusion using 10 - 0 nylon suture in refractory glaucoma: a case report. BMC Ophthalmol. 2023;23(1):418.

30. Linton E, Au L. Technique of Xen Implant Revision Surgery and the Surgical Outcomes: A Retrospective Interventional Case Series. Ophthalmol Ther. 2020;9(1):149-57.

31. Sng CCA, Chew PTK, Htoon HM, Lun K, Jeyabal P, Ang M. Case Series of Combined XEN Implantation and Phacoemulsification in Chinese Eyes: One-Year Outcomes. Adv Ther. 2019;36(12):3519-29.

32. Rooney DM, Shadid HR, Siegel LI, Watnick RL, Lesser GR, Obertynski T, et al. Postoperative Complications of Ab Interno Gelatin Microstent. J Glaucoma. 2019;28(5):e77-e81.

33. Kerr NM, Wang J, Sandhu A, Harasymowycz PJ, Barton K. Ab Interno Gel Implant-associated Bleb-related Infection. Am J Ophthalmol. 2018;189:96-101.

34. Galimi ME, Weller JM, Kruse FE, Laemmer R. Risk factors for ocular hypotony after XEN Gel Stent implantation. Graefes Arch Clin Exp Ophthalmol. 2023;261(3):769-78.

35. Widder RA, Dietlein TS, Dinslage S, Kuhnrich P, Rennings C, Rossler G. The XEN45 Gel Stent as a minimally invasive procedure in glaucoma surgery: success rates, risk profile, and rates of re-surgery after 261 surgeries. Graefes Arch Clin Exp Ophthalmol. 2018;256(4):765-71.

36. Hengerer FH, Kohnen T, Mueller M, Conrad-Hengerer I. Ab Interno Gel Implant for the Treatment of Glaucoma Patients With or Without Prior Glaucoma Surgery: 1-Year Results. J Glaucoma. 2017;26(12):1130-6.

37. Wanichwecharungruang B, Ratprasatporn N. 24-month outcomes of XEN45 gel implant versus trabeculectomy in primary glaucoma. PLoS One. 2021;16(8):e0256362.

38. Smith M, Charles R, Abdel-Hay A, Shah B, Byles D, Lim LA, et al. 1-year outcomes of the Xen45 glaucoma implant. Eye (Lond). 2019;33(5):761-6.

39. Tan SZ, Walkden A, Au L. One-year result of XEN45 implant for glaucoma: efficacy, safety, and postoperative management. Eye (Lond). 2018;32(2):324-32.

40. Karimi A, Lindfield D, Turnbull A, Dimitriou C, Bhatia B, Radwan M, et al. A multi-centre interventional case series of 259 ab-interno Xen gel implants for glaucoma, with and without combined cataract surgery. Eye (Lond). 2019;33(3):469-77.

41. Sacchi M, Fea AM, Monsellato G, Tagliabue E, Villani E, Ranno S, et al. Safety and Efficacy of Ab Interno XEN 45 Gel Stent in Patients with Glaucoma and High Myopia. J Clin Med. 2023;12(7).

42. Cutolo CA, Negri L, Olivari S, Cappelli F, Traverso CE, Iester M. Choroidal Detachment after XEN Gel Stent Implantation. J Ophthalmol. 2021;2021:6674505.

43. Schlenker MB, Ong JA, Wu P, Jinapriya D, Zack B, Dorey MW, et al. Surgeon Experience as a Risk Factor for Short-Term Failure for Ab Interno Gelatin Microstent: A Canadian Multicenter Propensity-Matched Study. Ophthalmol Glaucoma. 2022;5(1):67-76.

44. Grover DS, Flynn WJ, Bashford KP, Lewis RA, Duh YJ, Nangia RS, et al. Performance and Safety of a New Ab Interno Gelatin Stent in Refractory Glaucoma at 12 Months. Am J Ophthalmol. 2017;183:25-36.

45. Reitsamer H, Sng C, Vera V, Lenzhofer M, Barton K, Stalmans I, et al. Two-year results of a multicenter study of the ab interno gelatin implant in medically uncontrolled primary open-angle glaucoma. Graefes Arch Clin Exp Ophthalmol. 2019;257(5):983-96.

46. Galal A, Bilgic A, Eltanamly R, Osman A. XEN Glaucoma Implant with Mitomycin C 1-Year Follow-Up: Result and Complications. J Ophthalmol. 2017;2017:5457246.

47. Sng CC, Wang J, Hau S, Htoon HM, Barton K. XEN-45 collagen implant for the treatment of uveitic glaucoma. Clin Exp Ophthalmol. 2018;46(4):339-45.
